# Integrated network pharmacology and cellular assay reveal the biological mechanisms of *Limonium sinense* (Girard) Kuntze against Breast cancer

**DOI:** 10.1186/s12906-023-04233-z

**Published:** 2023-11-13

**Authors:** Hualong Zhao, Siyuan Wang, Philip T.F. Williamson, Rob M. Ewing, Xinhui Tang, Jialian Wang, Yihua Wang

**Affiliations:** 1 School of Marine and Biological Engineering, Yancheng Teachers’ University, Xiwang Road, Yancheng, 224002 PR China; 2 Biological Sciences, Faculty of Environmental and Life Sciences, University of Southampton, Southampton, SO17 1BJ UK; 3 Institute for Life Sciences, University of Southampton, Southampton, SO17 1BJ UK

**Keywords:** *Limonium Sinense* (Girard) Kuntze, Network pharmacology, Breast cancer, RNA-sequencing, Apigenin

## Abstract

**Background:**

*Limonium Sinense *(Girard) Kuntze (*L. sinense*) has been widely used for the treatment of anaemia, bleeding, cancer, and other disorders in Chinese folk medicine. The aim of this study is to predict the therapeutic effects of *L. sinense* and investigate the potential mechanisms using integrated network pharmacology methods and in vitro cellular experiments.

**Methods:**

The active ingredients of *L. sinense* were collected from published literature, and the potential targets related to *L. sinense* were obtained from public databases. Gene Ontology (GO), Kyoto Encyclopedia of Genes and Genomes (KEGG) and DisGeNET enrichment analyses were performed to explore the underlying mechanisms. Molecular docking, cellular experiments, RNA-sequencing (RNA-seq) and Gene Expression Omnibus (GEO) datasets were employed to further evaluate the findings.

**Results:**

A total of 15 active ingredients of *L. sinense* and their corresponding 389 targets were obtained. KEGG enrichment analysis revealed that the biological effects of *L. sinense* were primarily associated with “Pathways in cancer”. DisGeNET enrichment analysis highlighted the potential role of *L. sinense* in the treatment of breast cancer. Apigenin within *L. sinense* showed promising potential against cancer. Cellular experiments demonstrated that the *L. sinense* ethanol extract (LSE) exhibited a significant growth inhibitory effect on multiple breast cancer cell lines in both 2D and 3D cultures. RNA-seq analysis revealed a potential impact of LSE on breast cancer. Additionally, analysis of GEO datasets verified the significant enrichment of breast cancer and several cancer-related pathways upon treatment with Apigenin in human breast cancer cells.

**Conclusion:**

This study predicts the biological activities of *L. sinense* and demonstrates the inhibitory effect of LSE on breast cancer cells, highlighting the potential application of *L. sinense* in cancer treatment.

**Supplementary Information:**

The online version contains supplementary material available at 10.1186/s12906-023-04233-z.

## Introduction

*Limonium sinense* (Girard) Kuntze (*L. sinense*), also known as *Latouchea Fokiensis* and *Limoniumspp*, is an endemic plant species with important commercial value in Chinese medicine [1]. *L. sinense* mainly grows in the seashores and salt marshes regions of mainland China, western Taiwan, and Ryukyus islands in Japan [2, 3]. In Chinese folk medicine, *L. sinense* is commonly used for the treatment of bleeding, fever, hepatitis, haemostasis, anaemia, menorrhagia, irregular menstruation, and other disorders [4, 5]. Studies have reported that polysaccharides derived from the root of *L. sinense* exhibit potent anti-tumour and immunomodulatory activities with low toxicity, effectively inhibiting the growth of tumours in mice [6]. Notably, LSP21, a polysaccharide separated from crude *L. sinense* polysaccharides, has demonstrated remarkable anti-tumour effects by inhibiting cell proliferation and inducing cell death [7]. Furthermore, *L. sinense* extracts have shown hepatoprotective activity against carbon tetrachloride and D-galactosamine intoxication in rats, with the underlying mechanism being associated with mitochondrial protection [2, 8, 9]. Other studies revealed significant anti-viral activity of *L. sinense*, and compounds such as gallic acid, samarangenin B and myricetin derived from *L. sinense* exhibited significant inhibitory effects against viral infections [10–12]. Our previous study [13] has found a significant inhibitory effect of the water extract of *L. sinense* on multiple types of human breast cancer cells. However, further underlying mechanisms of the pharmacological activities of *L. sinense* still remain to be elucidated.

**Fig. 1:**
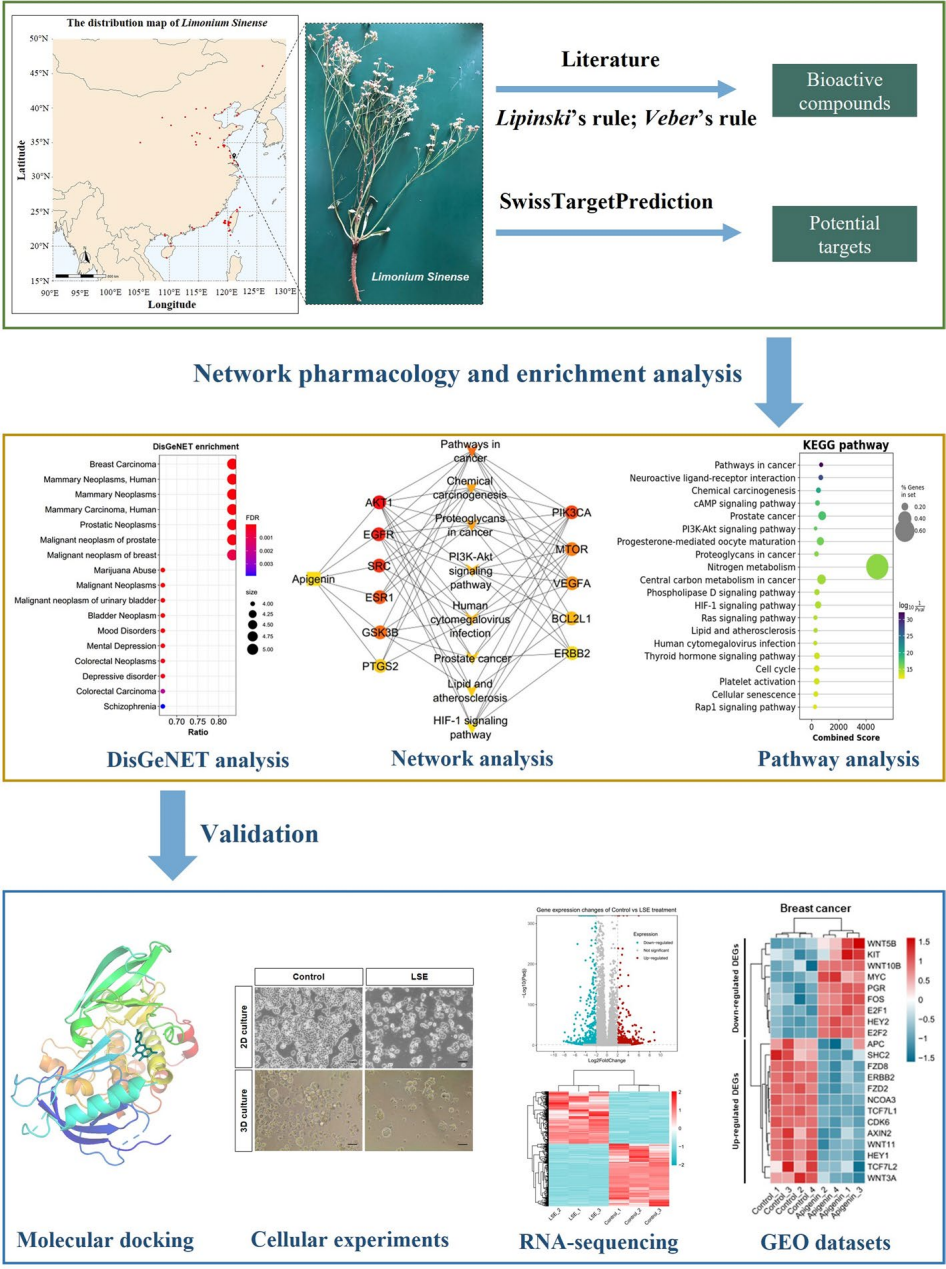
Flow chart of the study

More than 40 chemical compounds have been identified from *L. sinense*, most of which are flavonoids [12, 14]. However, there are few studies investigating the biological activities of these active ingredients and their associated mechanisms in *L. sinense.* In this study, network pharmacology and in vitro cellular experiments were employed to gain insights into the molecular basis of the therapeutic effects of *L. sinense*. We first explored the active ingredients and possible targets of *L. sinense*. By constructing the Compound-Target-Pathway network, a hub connection was identified from the network. Molecular docking, cellular experiments, RNA-sequencing (RNA-seq) and Gene Expression Omnibus (GEO) datasets were further performed to verify earlier findings. The detailed technical strategy of this study is shown in Fig. 1.

## Materials and methods

### Collection and preparation of sample material

Plant collection and extraction were carried out as previously reported [13]. Healthy whole plants of *L. sinense* were collected from the coastal region in Jiangsu, eastern China (33°09′33.0" N, 120°46′40.4" E). The collection of *L. sinense* was conducted with the oral approval of local authorities and in full compliance with China’s biodiversity rights and regulations. No specific license was required for the collection of *L. sinense*.

The whole plants were washed, and subsequently oven-dried at 60 °C until the weight was constant. After drying, the plants were crushed and extracted with 95% ethanol using a sonicator (Fisher Scientific, UK) at 1:400 (m/v) ratio with a processing time of 50 seconds and an elapsed time of 20 seconds, for a total extraction time of 30 minute. The extract process was repeated 3 times under the same conditions. The extracts were then put to suction filtration, rotary evaporated, and freeze-dried to powder. The powder was aliquoted and stored at -20 °C until future use when extracts were diluted with dimethylsulfoxide (DMSO) and filtered through a 0.45 μm filter (Millipore filter membranes, Merck, UK).

### Screening of active compounds and targets of *L. sinense*

*L. sinense* is not included in the Traditional Chinese Medicine Systems Pharmacology Database and Analysis Platform (TCMSP) or the Encyclopedia of Traditional Chinese Medicine (ETCM) platform, which allow users to explore the relationships or build networks among drugs, targets and diseases [15, 16]. The compounds of *L. sinense* were therefore obtained from the published literature. Active compounds were screened using the SwissADME database (http://swissadme.ch), based on the *Lipinski*’s rule of five [17] and *Veber*’s rule [18]. Then, the corresponding targets of the active compounds of *L. sinense* were obtained from SwissTargetPrediction.

### Construction of Protein–Protein interaction (PPI) network

The STRING (https://cn.string-db.org) database was utilized to analyse protein–protein interaction data. The species were limited to “Homo sapiens”, and medium confidence value > 0.4 was selected as the minimum required interaction score to construct the Protein–Protein interaction (PPI) network [19]. Then, the obtained PPI network from the STRING database was visualized by using Cytoscape *v3.9.1* software. The core nodes were selected based on the median values of three parameters in the interaction network: “Degree” which indicates the number of links to one node and reflects how often one node interacts with other nodes [20], “Betweenness Centrality” which quantifies the extent to which a node lies on paths between other nodes [21], and “Closeness Centrality” which measures the average distance from a node to other nodes [22]. The level of the three parameters represents the topological importance of the nodes in the interaction network, with more important nodes outputting higher values in the network [23].

### Enrichment analysis

Kyoto Encyclopedia of Genes and Genomes (KEGG) pathway enrichment analysis [24–26] was generated through GSEApy package, a comprehensive package for performing gene set enrichment analysis within the Python environment [27]. The GSEApy source code is freely available at https://github.com/zqfang/GSEApy. Detailed information regarding the specific GSEApy codes employed in this study can be found in the Supplementary methods section of the article. Diseases associated with the hub targets were enriched by using the disgenet2r package (*v0.99.3*) in R [28], the specific R codes for performing the enrichment analysis of disgenet2r package are available in the Supplementary methods section of the article. The gene ontology (GO) Biological Process term analysis was visualized using Cytoscape *v3.9.1* software. Groupings were facilitated by the Cytoscape AutoAnnotate plugin [29]. The GO term fusion was selected based on a statistical significance threshold of *P* ≤ 0.05.

### Network construction

In this process, network construction was performed as follows: First, the Compound-Target network (CT network) was built based on the active compounds of *L. sinense* and their potential targets. Next, the Pathway-Target network (PT network) was constructed by selecting the top 20 enriched KEGG pathways and their associated targets. Finally, a Compound-Target-Pathway network (CTP network) was established by integrating the CT network, PT network and the core targets identified from the PPI network. All visualized network graphs were created using Cytoscape *v3.9.1* software. The hub network was screened by using Cytohubba [30], a degree algorithm based Cytoscape plugin.

### Molecular docking

The crystal structure of AKT1 (PDB ID: 3O96), EGFR (PDB ID: 1XKK), SRC (PDB ID: 3EL8), ESR1 (PDB ID: 5ACC), GSK3B (PDB ID: 3I4B) and PTGS2 (PDB ID: 6COX) were downloaded from Protein Data Bank (https://www.rcsb.org/). Compound structure of Apigenin was downloaded from PubChem (https://pubchem.ncbi.nlm.nih.gov/). The docking studies were performed by PyRx Autodock VINA tool (*v0.8*). A grid box was set to cover the active site of crystal structure with a default exhaustiveness value of 8 [31]. The best compound with highest binding affinity (kcal/mol) was selected and visualized by using Discovery Studio (*version 2021 Client*) and PyMOL.

### Cell culture and reagents

Sources of cell lines and culture conditions were reported earlier [32–34]. HCC1806, HCC1395 and HCC1937 cells were maintained in Roswell Park Memorial Institute (RPMI) 1640 medium, (Gibco® by Life Technology) with 10% fetal bovine serum (FBS) and 1% (v/v) penicillin/streptomycin, (Gibco® by Life Technology). BT20, MDA-MB-157, MDA-MB-231 and MDA-MB-468 cell lines were maintained in Dulbecco’s modified Eagle’s medium (DMEM) (Gibco® by Life Technology) with 10% FBS and 1% (v/v) penicillin/streptomycin. All cells were kept at 37 °C and 5% CO_2_. No mycoplasma contamination was detected in the cell lines used. In 3D culture, cells were seeded in 96-well ultralow attachment plate in 100 µl at plating densities between 3,000 and 7,000 cells/well. Cells were cultured in 1:1 DMEM:F12, (Gibco® by Life Technology) media plus 1% P/S, 2% B27 (Gibco® by Life Technology), 20 ng/ml epidermal growth factor (EGF) (PEPROTECH) and 20 ng/ml basic fibroblast growth factor (bFGF) (PEPROTECH) at 37 °C and 5% CO_2_ for 14 days. After the incubation period, the images were taken using with × 40 magnification.

### Cell viability assay

Cell viability assays was performed as previously described [32]. Cells were plated into 96-well plate with a density of 8000 cells/well. CellTiter-Glo® Luminescent cell viability assay (Promega) was performed 48 h after treatment according to the manufacturer’s protocol using GloMax® Discover Microplate Reader (Promega). For cell viability in 3D cultures, 100 µl of CellTiter-Glo® reagent was added into each well and incubated at room temperature for 1 h, followed by measurement.

For IC_50_ a serial dilution starting at 125 μg/mL was made in assay buffer. IC_50_ values were derived by a dose–response (variable slope) curve using GraphPad Prism *v10.0.0* software. The reported data are average of at least three independent experiments.

### RNA-sequencing (RNA-seq) analysis

RNA isolation and mRNA sequencing of samples were performed following the manufacturer’s instructions (Novogene, UK) as previously described [35, 36]. The MDA-MB-468 cells were treated with LSE for 48 h. Total RNA was isolated using RNeasy mini kit (Qiagen) according to manufacturer’s instructions and quantified using a Nanodrop Spectophotometer 2000c (Thermo Fisher Scientific). A total amount of 3 μg RNA per sample was used as input material for library construction. Sequencing libraries were generated using NEBNext® UltraTM RNA Library Prep Kit for Illumina® (NEB, Ipswich, Massachusetts, USA) following manufacturer’s instruction. Libraries were pooled in equimolar and sequenced using the paired-end strategy (2 × 150) on the Illumina NovaSeq 6000 platform following the standard protocols (Novogene, UK). Raw read counts were imported into RStudio (*v4.2.0*) and analysed by using DESeq2 (*v3.17*) R package [37]. Transcripts with low abundance (under 10 counts across all samples) were removed. The RNA-Seq data have been deposited in the Gene Expression Omnibus (GEO) database (accession code GSE244469). The R codes were provided in the R Scripts in the supplementary materials. Genes with |Log2FoldChange| above 2 and *P*_adj_ values less than 0.05 were considered as differentially expressed genes (DEGs).

### GSEA hallmark analysis

The collection of hallmark gene sets generated from the GSEA (Gene Set Enrichment Analysis) software (*v4.1.0*) (with registration) [38, 39]. The normalized counts generated in DESeq2 were put into GSEA software, and the analysis used h.all.v2023.1.Hs.symbols.gmt [Hallmarks] gene set database with the settings of perform: 1000 permutations, collapse/remap to gene symbol—no_collapse (use dataset ‘as is’ in the original format), permutation type—gene_set, enrichment statistic—weighted, metric for ranking genes—Signal2Noise, gene list sorting mode—real, gene list ordering mode—descending, max size of gene sets—500, and min size of gene sets—10.

### GEO dataset analysis

Gene Expression Omnibus (GEO) dataset analysis was performed as previous described [13]. GEO datasets of human breast cancer cells treated with Apigenin were screened, by searching the keywords “(breast cancer) AND (Apigenin)” and publication dates before 01/05/2023 in the National Centre for Biotechnology Information (NCBI) GEO platform. We only included datasets that met the following criteria: 1) mRNA expression data; 2) Homo sapiens samples; 3) Breast cancer cells; 4) minimum 3 biological replicates. Duplicate datasets were removed. Datasets with fewer than 10,000 genes were excluded to balance the number of analysed genes and sample size. Microarray probe IDs were translated to gene symbols according to the GPL annotation files provided in the GEO database. Probes mapped to multiple gene symbols were removed and genes mapped to multiple probe IDs were summarized by calculating the mean. Codes are available upon request. Genes with *P* value less than 0.05 and ∣Log_2_FoldChange∣ above 1 were considered as differentially expressed genes (DEGs). DEGs of each GEO datasets were obtained by GEO2R in the GEO platform.

### Statistical analysis

Comparison of two groups was statistically calculated by two paired, unpaired Student’s *t* test in GraphPad Prism *v10.0.3* (GraphPad Software Inc, San Diego, CA). Two-way analysis of variance (ANOVA) with Dunnett’s multiple comparison test was used for multiple comparisons. Results were considered significant if *P* < 0.05, where *P < 0.05, **P < 0.01, ***P < 0.001.

## Results

### Identification of active compounds and targets of *L. sinense*

**Fig. 2:**
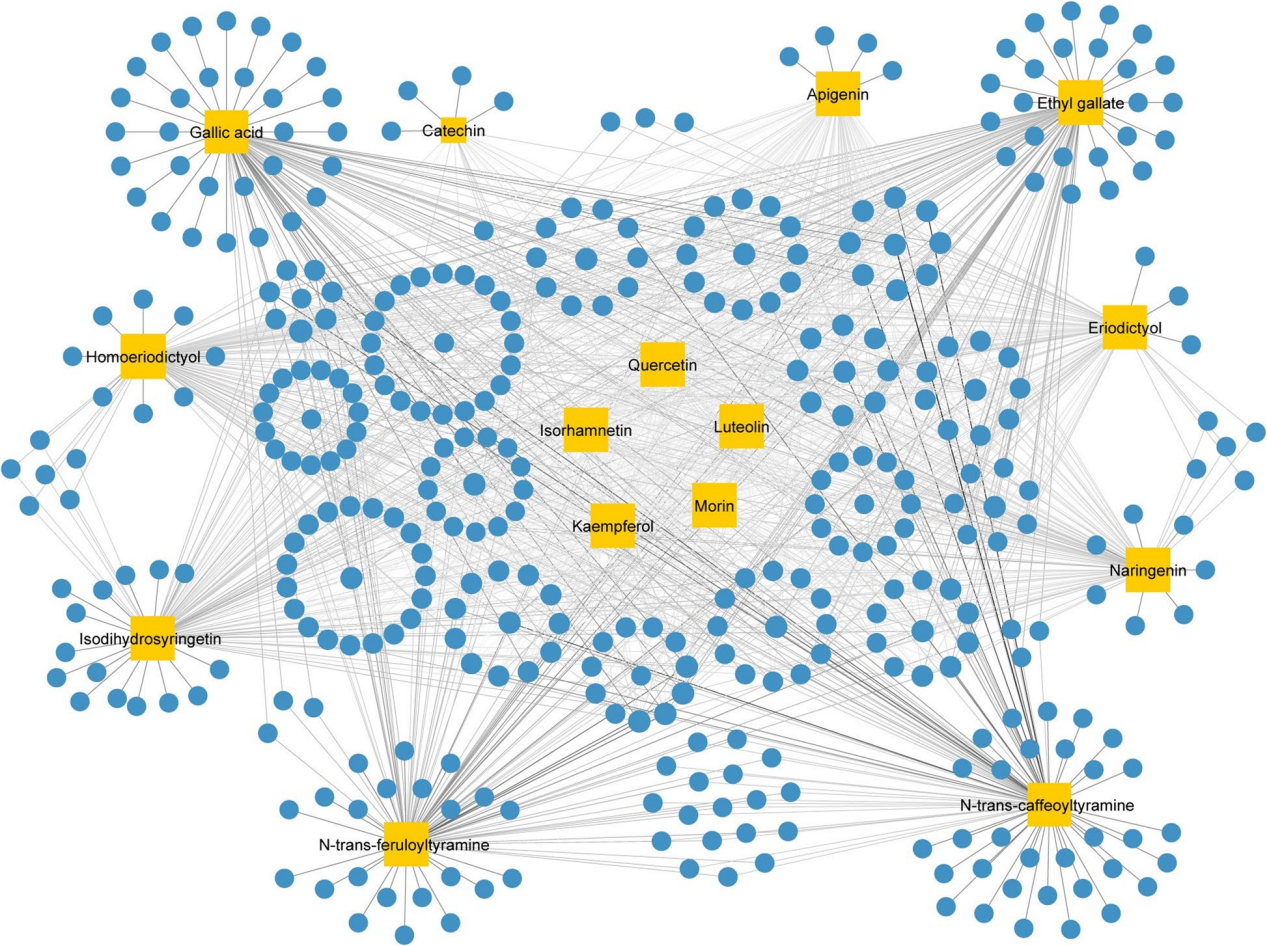
Compound-Target (CT) network of *L. sinense*. Graph consists of 15 compounds and 389 compound-related targets. The orange rectangles represent the small molecular components in *L. sinense*. The blue circles represent the relevant targets. The edges represent the relationship between compounds and target nodes. The node size is proportional to the node degree in the network, and the width and colour of the edges is proportional to the edge betweenness centrality

Based on the available published literature, a total of 42 natural compounds have been identified in *L. sinense* (Supplementary Table 1. The workflow for compound screening of *L. sinense* was shown in Supplementary Fig. 1). Out of these compounds, 15 have met the *Lipinski*’s rule of five and *Veber*’s rule, making them the active compounds of *L. sinense*. These active compounds include Gallic acid, Ethyl gallate, Apigenin, Naringenin, Luteolin, Kaempferol, Eriodictyol, ( +)-Catechin, N-trans-caffeoyltyramine, Quercetin, Morin, Homoeriodictyol, N-trans-feruloyltyramine, Isorhamnetin and Isodihydrosyringetin (Table 1). It is worth noting that the majority of these active compounds belong to the flavonoid group. Furthermore, 389 targets associated with these 15 active compounds were obtained after removing duplicate values (Supplementary Table 2). The Compound-Target network (CT network) revealed that all 15 bioactive compounds exhibited relatively high values of Degree, Betweenness Centrality (BC) and Closeness Centrality (CC) within the network (Fig. 2; Supplementary Table 3), showing significant connectivity and potential importance of these compounds in the overall network.

**Table 1:** Detailed information on active compounds in *L*. s*inense*

Compound Name	Compound Type	Molecular weight	nRB	nHA	nHD	TPSA	LogP
Gallic acid	Phenolic acid	170.12	1	5	4	97.99	-0.16
Ethyl gallate	Phenolic acid	198.17	3	5	3	86.99	0.49
Apigenin	Flavone	270.24	1	5	3	90	0.52
Naringenin	Flavanone	272.25	1	5	3	86.99	0.71
Luteolin	Flavone	286.24	1	6	4	111.13	-0.03
Kaempferol	Flavonol	286.24	1	6	4	111.13	-0.03
Eriodictyol	Flavanone	288.25	1	6	4	107.22	0.16
( +)-Catechin	Flavan-3-ol	290.27	1	6	5	110.38	0.24
N-trans-caffeoyltyramine	Alkaloid	299.32	6	4	4	89.79	1.65
Quercetin	Flavonol	302.24	1	7	5	131.36	-0.56
Morin	Flavonol	302.24	1	7	5	131.36	-0.56
Homoeriodictyol	Flavanone	302.28	2	6	3	96.22	0.41
N-trans-feruloyltyramine	Alkaloid	313.35	7	4	3	78.79	1.89
Isorhamnetin	Flavonol	316.26	2	7	4	120.36	-0.31
Isodihydrosyringetin	Flavanone	348.3	3	8	4	125.68	-0.66

*nRB* Number of Rotatable bonds, *optimal 0–10*, *nHA* Number of Hydrogen bond acceptors, optimal: 0–10; *nHD* Number of Hydrogen bond donors, optimal: 0–5, *TPSA* Topological Polar Surface Area, optimal: 0–140, *LogP* Log of the octanol/water partition coefficient, optimal: ≤ 5

### Construction of PPI network and the identification of core targets of *L. sinense*

**Fig. 3:**
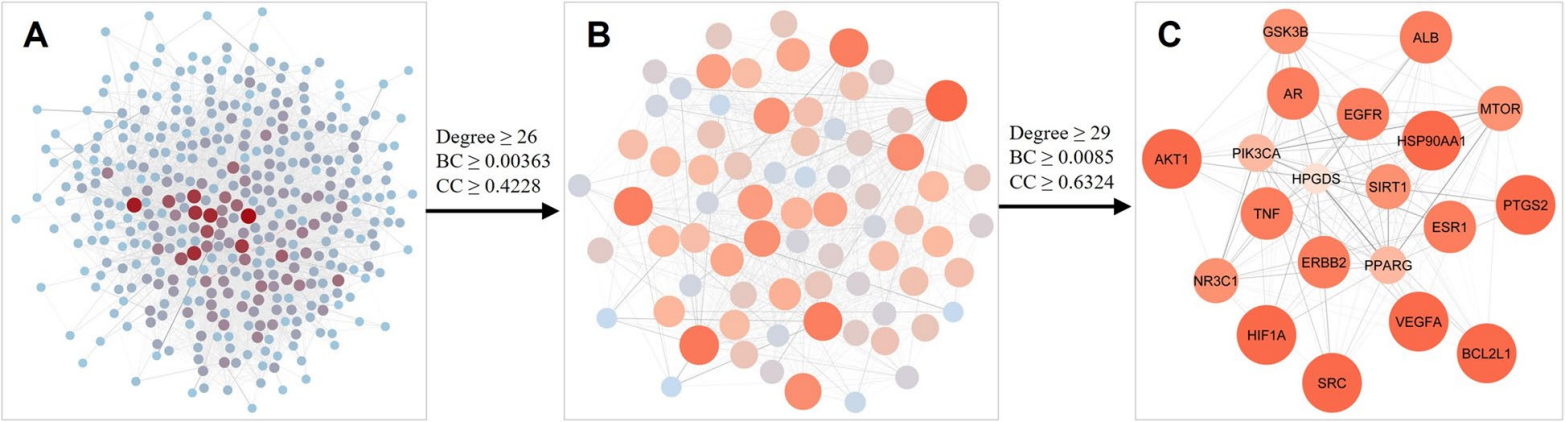
The process of topological screening for the PPI network. Each node represents a protein and each edge refers an interaction. The size and colour of the nodes represent the node degree in the network, and the width and colour of the edges represent the edge betweenness centrality. The core targets were screened based on the Degree, betweenness centrality (BC) and closeness centrality (CC) values in the network

A PPI network consisting of 389 nodes and 4,947 edges was established (Fig. 3A). Based on the threshold values of Degree, BC and CC, with the first screening using Degree ≥ 26, BC ≥ 0.00363 and CC ≥ 0.4228, the PPI network was pruned to 73 nodes and 1,049 edges (Fig. 3B). Subsequently, nodes with Degree ≥ 29, BC ≥ 0.0085 and CC ≥ 0.6324 were screened as the second screening threshold values and resulted in 20 core nodes and 178 edges (Fig. 3C). These 20 targets, namely *AKT1*, *SRC*, *ALB*, *EGFR*, *HSP90AA*, *ESR1*, *VEGFA*, *TNF*, *MTOR*, *HIF1A*, *ERBB2*, *AR*, *SIRT1*, *BCL2L1*, *PPARG*, *GSK3B*, *PTGS2*, *NR3C1*, *PIK3CA* and *HPGDS*, were defined as the core targets of *L. sinense* (Supplementary Table 4), which likely to contribute to its pharmacological activities.

### Enrichment analysis of compound targets

**Fig. 4:**
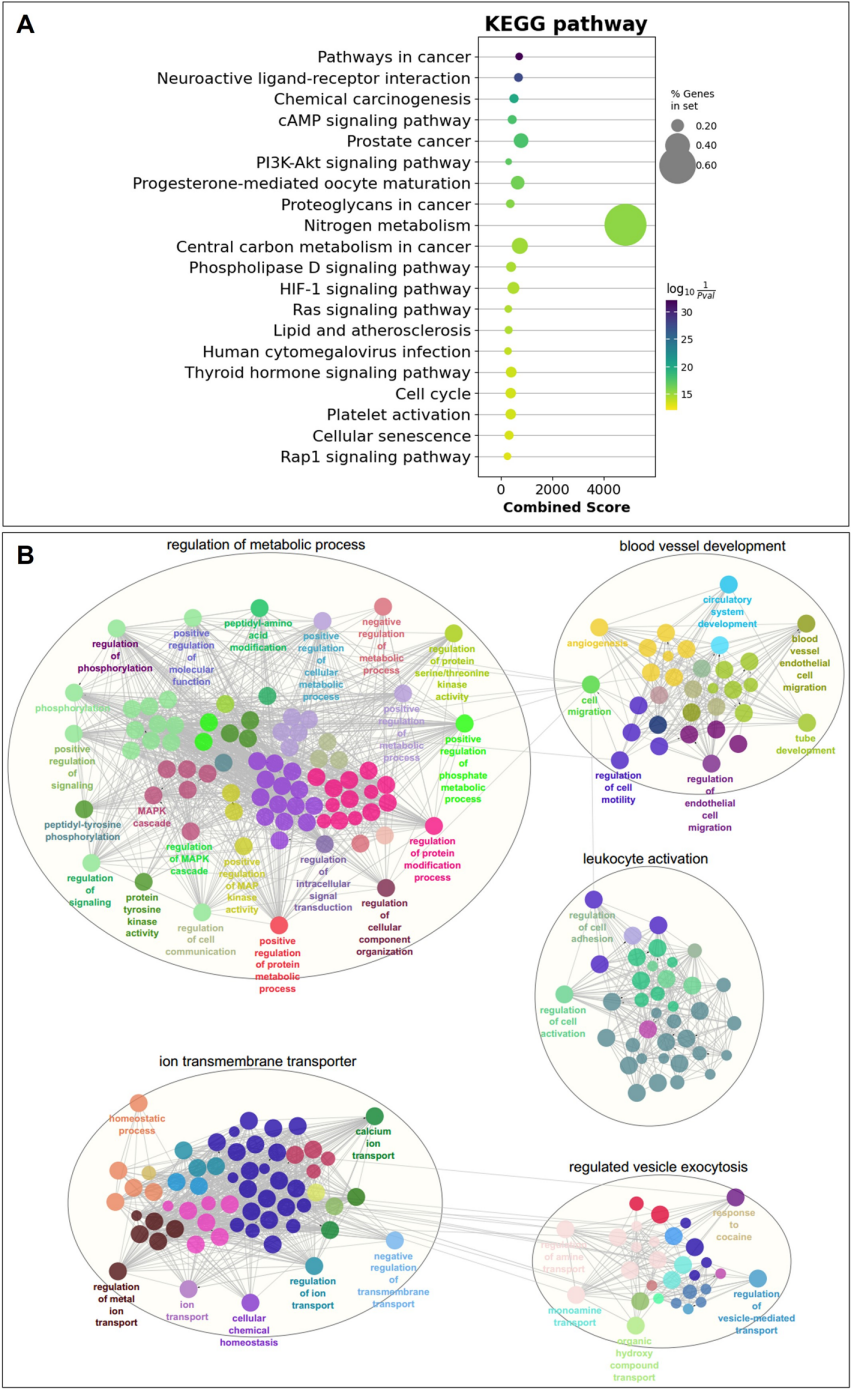
Enrichment analysis from *L. sinense* targets. **A** Scatter plot showing the Kyoko Encyclopedia of Genes and Genomes (KEGG) pathways (Top20) enriched by the *L. sinense* potential targets. The sizes of circles represent the percentage of genes in the gene set, and the colours of circles represent the log_10_($\frac{1}{Pvalue}$). Combined score is defined by the Enrichr. **B** ClueGO analysis of the Biological Process terms enriched by the *L. sinense* potential targets. The Biological Process terms were grouped by using the AutoAnnotate v.1.2 Cytoscape plugin, and networks showing the top 5 Biological Process clusters which involved in the regulation of metabolic process, ion transmembrane transporter, blood vessel development, leukocyte activation, and regulated vesicle exocytosis. Biological Process terms are represented as nodes, and the node size represents the term enrichment significance. Functionally grouped networks are linked to their biological function, where only the significant term (*P* ≤ 0.05) in the group is labelled

The enrichment analysis was retrieved 202 KEGG pathways on the 389 compound targets with a screening threshold of a *P* value less than 0.05 (Supplementary Table 5). Among the top-ranked enrichment results, several cancer-related pathways were significantly identified in the KEGG pathways, including Pathways in cancer, Chemical carcinogenesis, Prostate cancer, Proteoglycans in cancer and Central carbon metabolism in cancer (Fig. 4A).

Within the context of Gene Ontology (GO) terms, we identified a total of 1,109 Biological Process terms (Supplementary Table 6), which were further grouped into 178 clusters using the AutoAnnotate *v.1.2* Cytoscape plugin. Among these clusters, the most prominent ones included the regulation of metabolic process, ion transmembrane transporter, blood vessel development, leukocyte activation, and regulated vesicle exocytosis (Fig. 4B). Notably, the blood vessel development cluster encompassed terms such as angiogenesis, blood vessel development, tube development, and blood vessel morphogenesis, which could potentially relate to the blood-enriching properties of *L. sinense* (Supplementary Fig. 2). These findings suggest that *L. sinense* may play a role in the regulation of cancer and cancer-related pathways, offering insights into its potential mechanism for enhancing blood enrichment functions.

### CTP network construction and the identification of hub connections

**Fig. 5:**
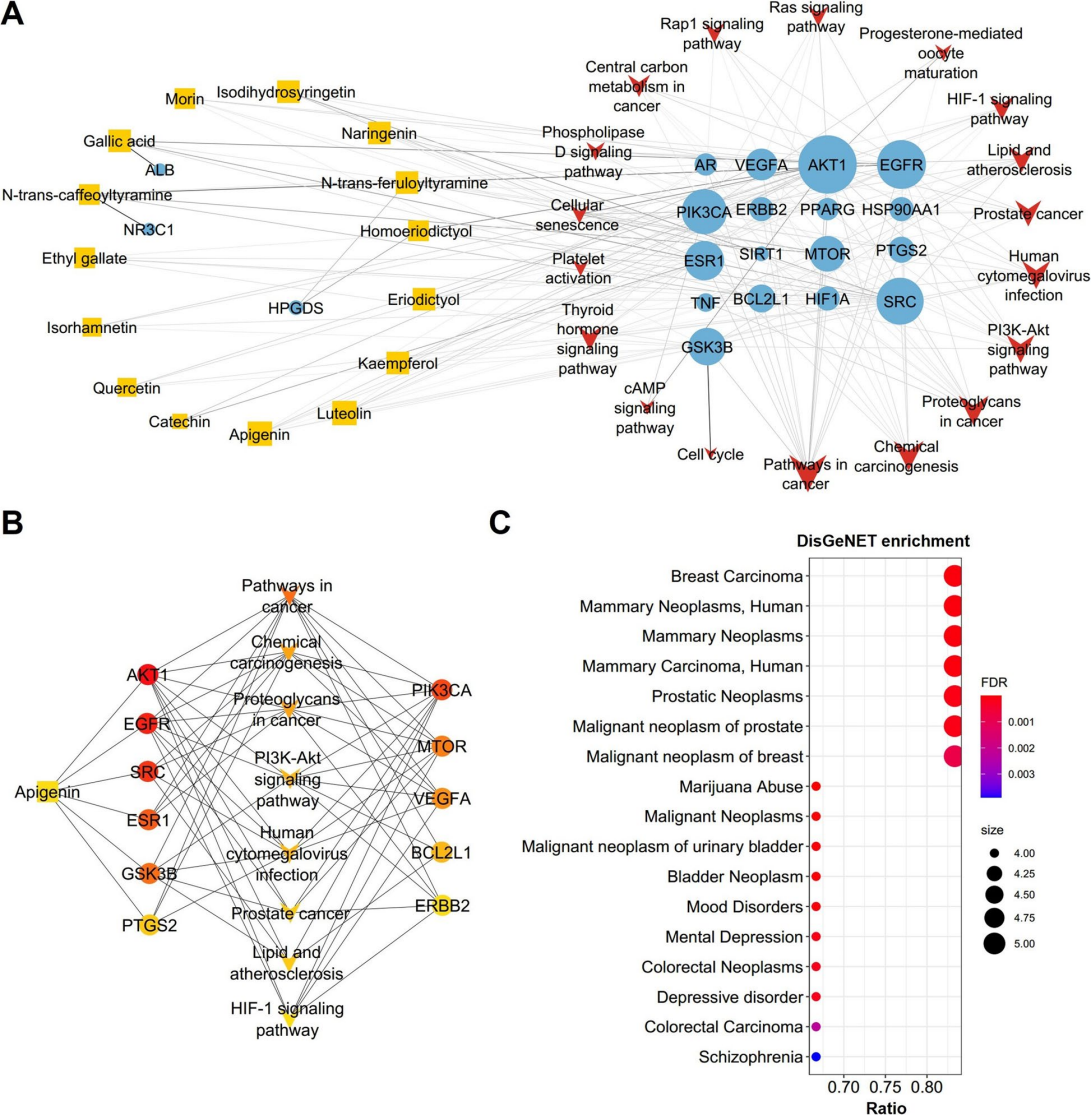
Hub network and DisGeNET enrichment analysis. **A** Compound-Target-Pathway network of *L. sinense*. The orange rectangles represent the small molecular components in *L. sinense*. The blue circles represent the relevant targets. The red V nodes represent the relevant signalling pathways. The edges represent the relationship among compounds, targets and pathways. The node size is proportional to the node degree in the network, and the width and colour of the edges is proportional to the edge betweenness centrality. **B** Hub network identified from the CTP network using the CytoHubba. Colour node indicates the degree value of each node in the network. **C** DisGeNET enrichment analysis of hub targets. The size of the dots refers to the number of genes enriched for that disease (the greater the number of genes, the larger the dot). The FDR is defined by a colour scale, the closer it is to red, the greater the FDR value and the greater the association

The top 20 KEGG pathways and their associated targets were selected to construct the TP network (Supplementary Fig. 3). Subsequently, a CTP network was built by incorporating the 15 active compounds, 20 core targets and the top 20 KEGG pathways, and in the end the CTP network consisted of 53 nodes and 195 edges (Fig. 5A). A hub network was identified from the CTP network which contains 20 nodes and 65 edges (Fig. 5B, Supplementary Table 7). Within the hub network, 6 hub targets, namely *AKT1*, *EGFR*, *SRC*, *ESR1* and *GSK3B*, were found to be directly regulated by Apigenin. Additionally, 8 signalling pathways were involved in the hub network. Many of these signalling pathways were related with cancer processes, including Pathways in cancer, Chemical carcinogenesis, Proteoglycans in cancer and Prostate cancer. Furthermore, HIF-1 signalling pathway was also identified in the hub network, indicating a crucial role of HIF-1 signalling pathway in the biological activities of *L. sinense*.

To investigate the associated diseases with the hub targets, an analysis of the DisGeNET database (https://www.disgenet.org/ accessed on 15 May 2023) (*version 7.0*) was performed via the disgenet2r package (*v0.99.3*) in R. The results revealed that the top enriched diseases were primarily related to breast cancer, such as Breast carcinoma, Mammary neoplasms, and Mammary carcinoma (Fig. 5C; Supplementary Table 8). These findings suggest a potential therapeutic role of *L. sinense* in the treatment of breast cancer.

### Molecular docking between Apigenin and hub targets

**Fig. 6:**
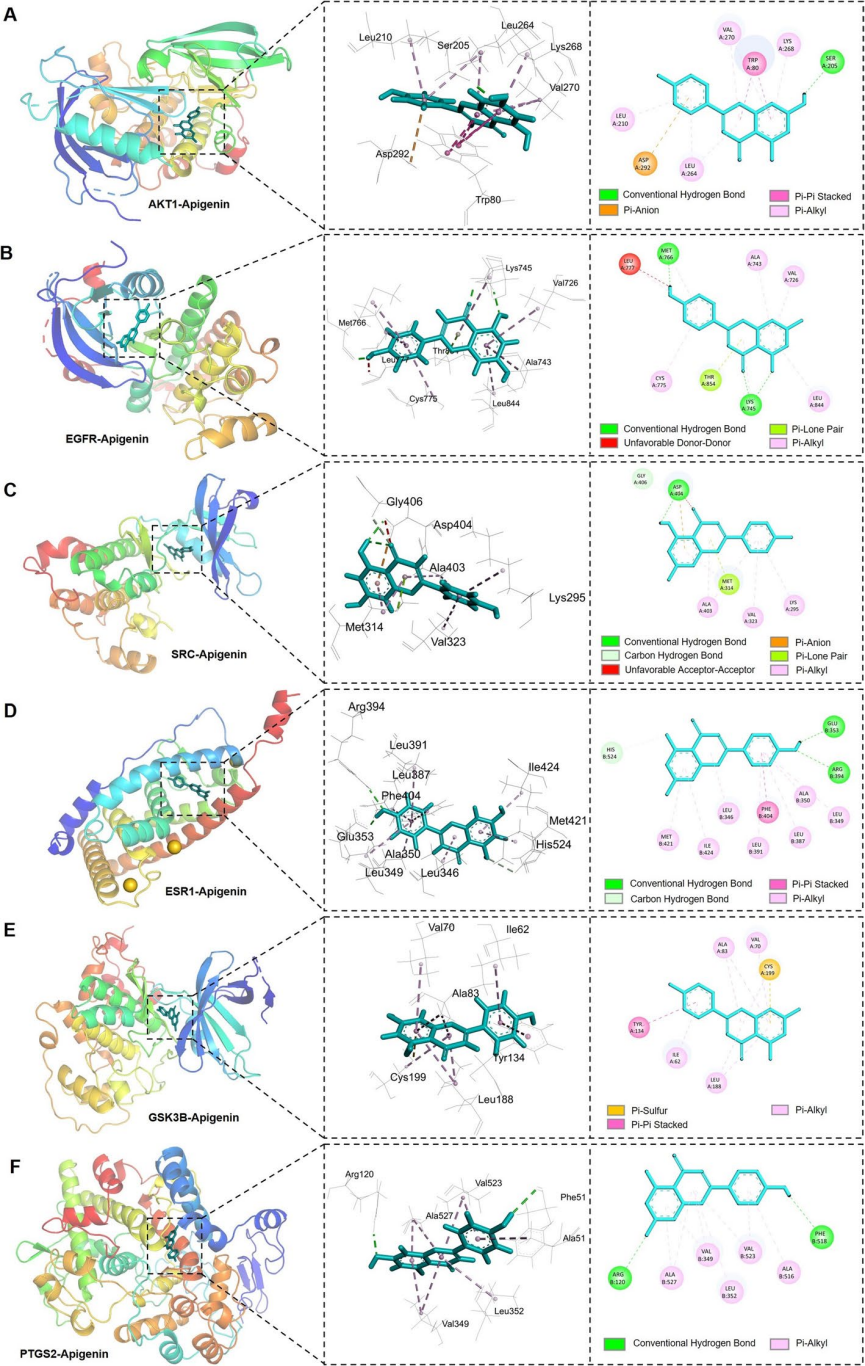
Molecular docking analysis of Apigenin with hub target proteins. **A-F** Graphs showing the binding mode and molecular interactions of Apigenin with AKT1, EGFR, SRC, ESR1, GSK3B and PTGS2, respectively. The 3D graphs showing the binding model of Apigenin and each target. The 2D graphs showing the specific interactions between Apigenin and the hub targets

Molecular docking was employed to validate the interactions between Apigenin and 6 hub target proteins. The results presented that Apigenin exhibited a strong molecular docking affinity with 6 hub targets, as evidenced by docking scores of less than -5 kcal/mol (Table 2). AKT1 displayed the best binding activity with Apigenin (affinity = -9.6), followed by ESR1(affinity = -8.9), PTGS2 (affinity = -8.8), SRC (affinity = -8.6), EGFR (affinity = -8.5), and GSK3B (affinity = -8.4). The binding structures showed that Apigenin deeply entered the binding sites of each hub target, with abundant hydrogen-bond donors and acceptors around the binding cavity (Fig. 6). The binding sites and interaction bonds of Apigenin with each hub target protein were shown in Table 2. Taking AKT1 as an example, Apigenin bound to site of Ser205 in AKT1 via conventional hydrogen bond, while bound to sites of Asp292, Trp80, Leu210, Leu264, Lys268 and Val270 in AKT1 through pi-anion, pi-pi stacked and pi-alkyl bonds (Fig. 6A).

**Table 2:** Binding affinities and Receptor-ligand interactions of Apigenin with hub targets

Compound name	Protein	Binding affinity (kcal/mol)	Interaction residues	Interaction bonds
Apigenin	AKT1	-9.6	Ser205, Asp292, Trp80, Leu210, Leu264, Val270, Lys268	Conventional Hydrogen Bond, Pi-Anion, Pi-Pi Stacked, Pi-Alkyl
	EGFR	-8.5	Lys745, Met766, Thr854, Leu777, Val726, Ala743, Cys775, Leu844	Conventional Hydrogen Bond, Unfavorable Donor-Donor, Pi-Lone Pair, Pi-Alkyl
	SRC	-8.6	Asp404, Gly406, Met314, Lys295, Val323, Ala403	Conventional Hydrogen Bond, Carbon Hydrogen Bond, Unfavorable Acceptor-Acceptor, Pi-Anion, Pi-Lone Pair, Pi-Alkyl
	ESR1	-8.9	Glu353, Arg394, His524, Phe404, Leu346, Leu349, Ala350, Leu387, Leu391, Met421, Ile424	Conventional Hydrogen Bond, Carbon Hydrogen Bond, Pi-Pi Stacked, Pi-Alkyl
	GSK3B	-8.4	Cys199, Tyr134, Ile62, Val70, Ala83, Leu188	Pi-Sulfur, Pi-Pi Stacked, Pi-Alkyl
	PTGS2	-8.8	Arg120, Phe518, Val349, Leu352, Ala516, Val523, Ala527	Conventional Hydrogen Bond, Pi-Alkyl

### Effects of *L. sinense* ethanol extract (LSE) on breast cancer cells

In our previous study, we demonstrated that the water extract of *L. sinense* has a significant inhibitory effect on the growth of multiple breast cancer cell lines [13]. Based on the network pharmacology results, we found that Apigenin may also play a role against cancer. These represent a new class of compounds, as unlike in our previous study, the Apigenin is almost insoluble in water and is present in the ethanol extract [40], we therefore chose ethanol as the solvent to extract compounds from *L. sinense* in this analysis.

**Fig. 7:**
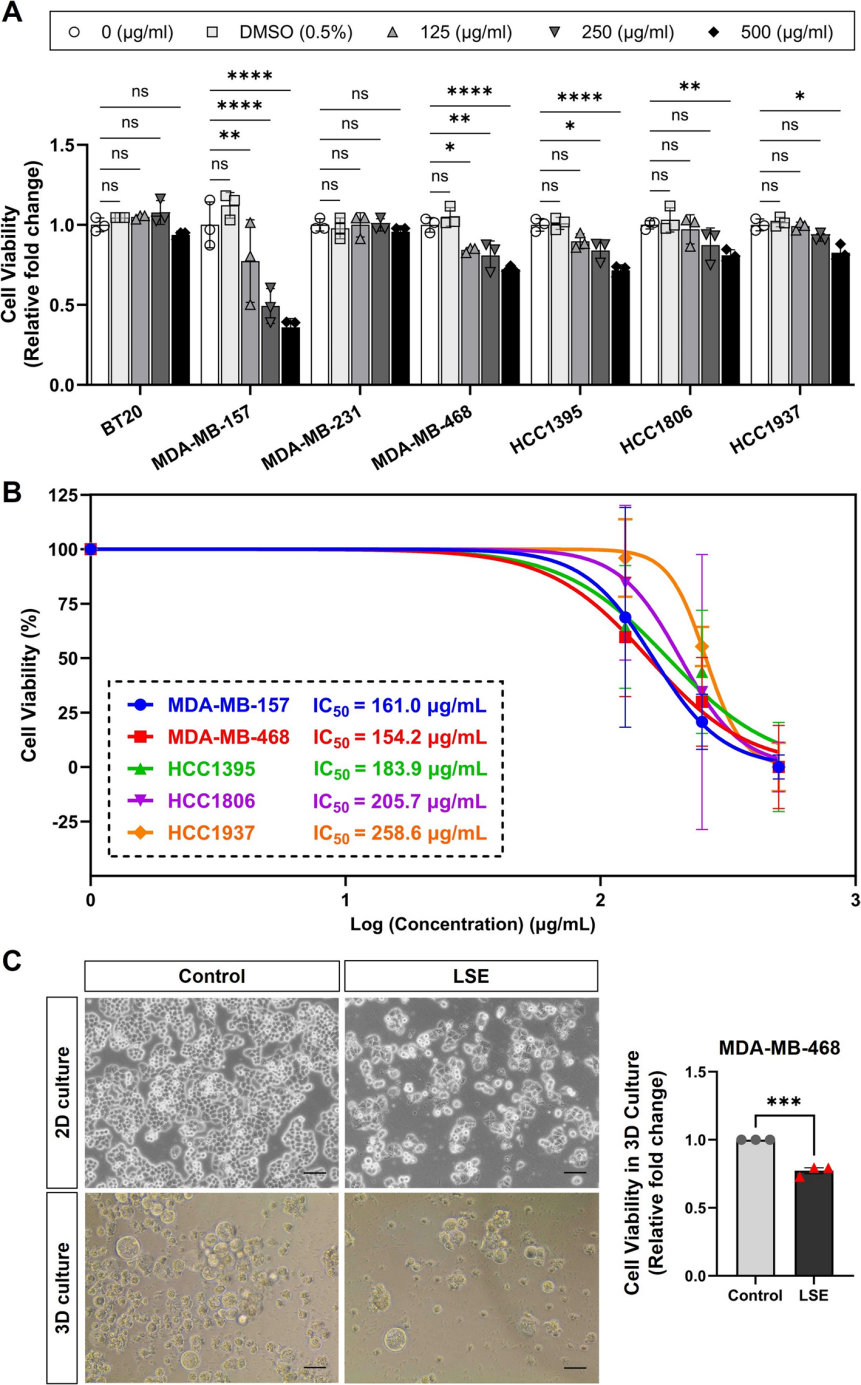
Cell viability assay of ethanol extract of *L. sinense* (LSE) on breast cancer cells. **A** Graph showing relative cell viability in multiple cell lines treated with *L. sinense* ethanol extract (LSE) at the indicated concentration for 48 h. Cell-Titer Glo® assay was performed to measure cell viability. Data are mean ± SD; n = 3 samples per group. ns, not significant; *P < 0.05; **P < 0.01 and ***P < 0.001 by the Two-way ANOVA. **B** IC_50_ values of *L. sinense* ethanol extract (LSE) against tested breast cancer cell lines. IC_50_ values were derived by a dose–response (Variable slope) curve using GraphPad Prism software. Dose–response data points represent the mean value of three independent experiments. **C** Representative phase contrast microscopy images showing the morphology change of MDA-MB-468 cells treatment with LSE in 2D and 3D cultures. Scale bar: 50 µm. Bar plot showing the relative cell viability change (Cell-Titer Glo® assay) of MDA-MB-468 cells with indicated treatment in 3D culture. Data are mean ± SD. ns, not significant; *P < 0.05; **P < 0.01 by the Student's t-test

To study the effect of *L. sinense* ethanol extract (LSE) on breast cancer, 7 human breast cancer cell lines (BT20, MDA-MB-157, MDA-MB-231, MDA-MB-468, HCC1395, HCC1806 and HCC1937) were treated with LSE followed by a cell viability assay. The results showed that addition of LSE led to a strong inhibition of growth in multiple tested cell lines in a dose-dependent manner after 48 h treatment (Fig. 7A). The determination of IC_50_ values for each breast cancer cell line treated with LSE revealed significant inhibitory potency, particularly with notable effects observed on MDA-MB-468 cells (Fig. 7B). Furthermore, to confirm the effects of LSE on cell viability, a 3D mammosphere assay was performed on MDA-MB-468 cells. The results demonstrated a significant decrease in cell viability (*P* < 0.001) in LSE-treated MDA-MB-468 cells (Fig. 7C). These experiments provide further evidence of the growth inhibitory effect of *L. sinense* on breast cancer.

### Global transcriptomic changes in MDA-MB-468 cells exposed to LSE

**Fig. 8:**
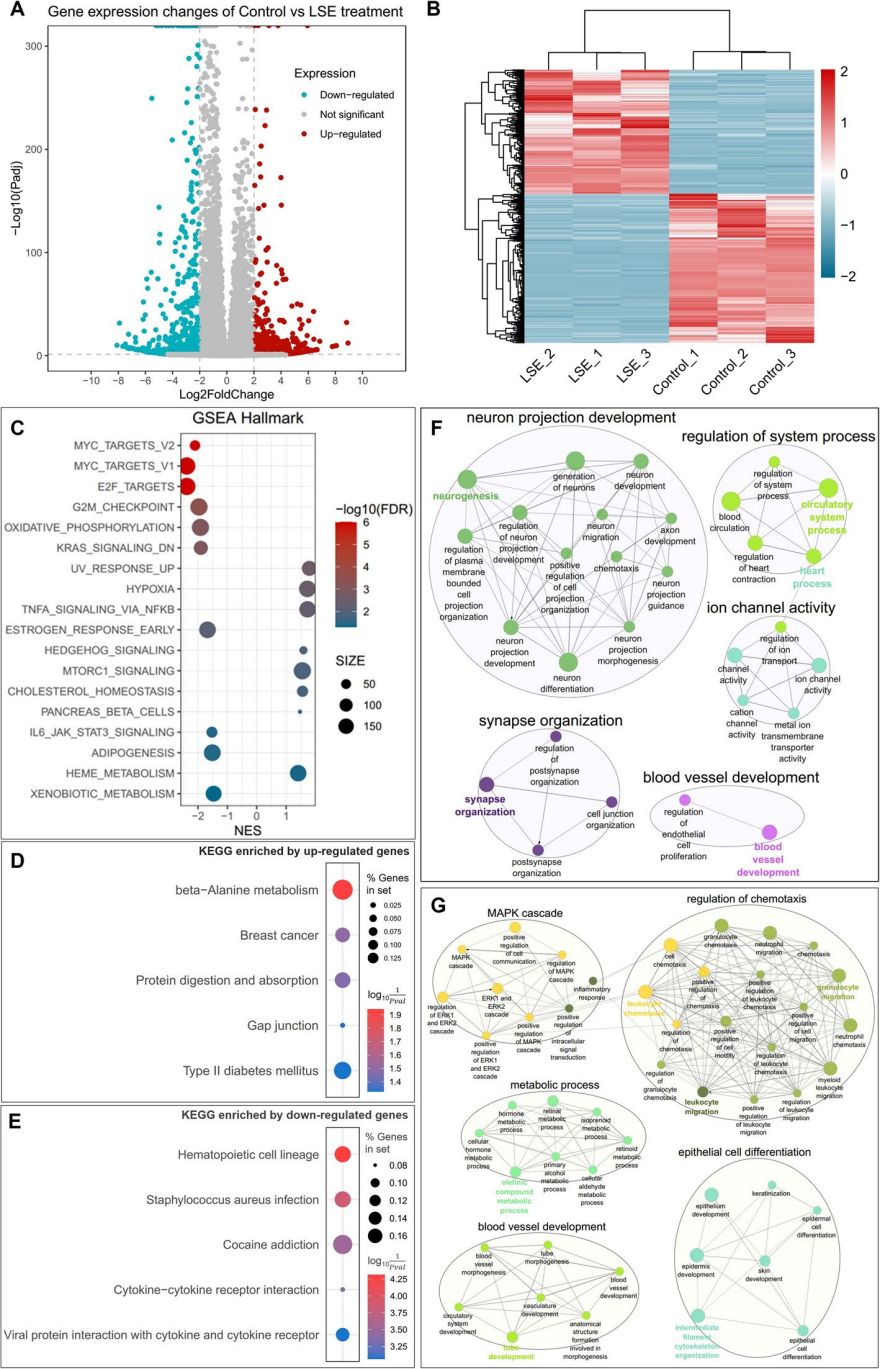
RNA sequencing (RNA-seq) analysis results and function enrichment analysis of differentially expressed genes (DEGs) in MDA-MB-468 cells treated with LSE. **A** Volcano plot showing the up- and down-regulated genes in LSE-treated MDA-MB-468 cells. Log2FoldChange in *x*-axis and -log_10_(*P*_adj_) in *y*-axis. Red indicates up-regulation, cyan down-regulation and gray not significant. **B** Heatmap showing DEGs in LSE-treated MDA-MB-468 cells. DEGs were selected based on a *P*_adj_ value less than 0.05 and a ∣Log2FoldChange∣ value greater than 2. **C** Scatter plot showing Gene Set Enrichment Analysis (GSEA) in MDA-MB-468 cells treated with LSE. The sizes of circles represent gene count, which is the number of genes in the gene set after filtering out those genes not in the expression dataset. The colours of circles represent the -log10 of the false discovery rate (FDR) values. **D** and **E** Scatter plots showing enriched Kyoto Encyclopedia of Genes and Genomes (KEGG) pathways from up- and down-regulated DEGs in MDA-MB-468 cells treated with LSE. The sizes of circles represent the percent of genes in set, and the colours of circles represent the indicates the log_10_($\frac{1}{Pvalue}$). Functionally grouped networks showing the top 5 Biological Process clusters enriched by the up-regulated (**F**) and down-regulated (**G**) DEGs in MDA-MB-468 cells treated with LSE. Biological Process terms are represented as nodes, and the node size represents the term enrichment significance. The networks are linked to their biological function, where only the significant term (*P* ≤ 0.05) in the group is labelled

To assess the cellular response to LSE unbiasedly, we characterized the global transcriptomic changes in MDA-MB-468 cells exposed to LSE by utilizing RNA sequencing (RNA-Seq). Principal component analysis (PCA) clearly demonstrated a distinct separation between control and LSE-treated samples (Supplementary Fig. 4). Differentially expressed genes (DEGs) were identified based on an adjusted *P* value (*P*_adj_) less than 0.05 and an absolute ∣Log2FoldChange∣ greater than 2, resulting in a total of 1,306 DEGs, with 594 up-regulated and 712 down-regulated genes (Fig. 8A; Supplementary Table 9). The hierarchical clustering showed that DEGs were grouped into 2 major clusters (Fig. 8B).

Subsequently, we performed pathway enrichment analysis using Gene Set Enrichment Analysis (GSEA) (Fig. 8C; Supplementary Table 10), Kyoto Encyclopedia of Genes and Genomes (KEGG) pathway analysis (Fig. 8D, E; Supplementary Table 11) and Gene Ontology (GO) enrichment analysis (Fig. 8F, G; Supplementary Table 12). Interestingly, Hypoxia (NES = 1.721, FDR = 0.003), Heme_metabolism (NES = 1.409, FDR = 0.042) and G2M_checkpoint (NES =-1.967, FDR = 0.0003) were significantly enriched. In the context of KEGG analysis, Breast cancer pathway (*P*-value = 0.032) was significantly enriched by the up-regulated genes. Furthermore, GO biological process analysis highlighted the significant enrichment of “blood vessel development” among both up- and down-regulated genes. These findings reinforce the potential therapeutic application of *L. sinense* in breast cancer treatment and underscore its role in enhancing blood enrichment functions.

### Effects of Apigenin on breast cancer cells

Finally, we utilized publicly available GEO datasets on human breast cancer cells with the treatment of Apigenin to gain further insights into the mechanisms of Apigenin against breast cancer. Two GEO datasets were selected based on predetermined screening criteria, which included a total of 14 samples derived from 2 different types of human breast cancer cell lines (Supplementary Table 13).

**Fig. 9:**
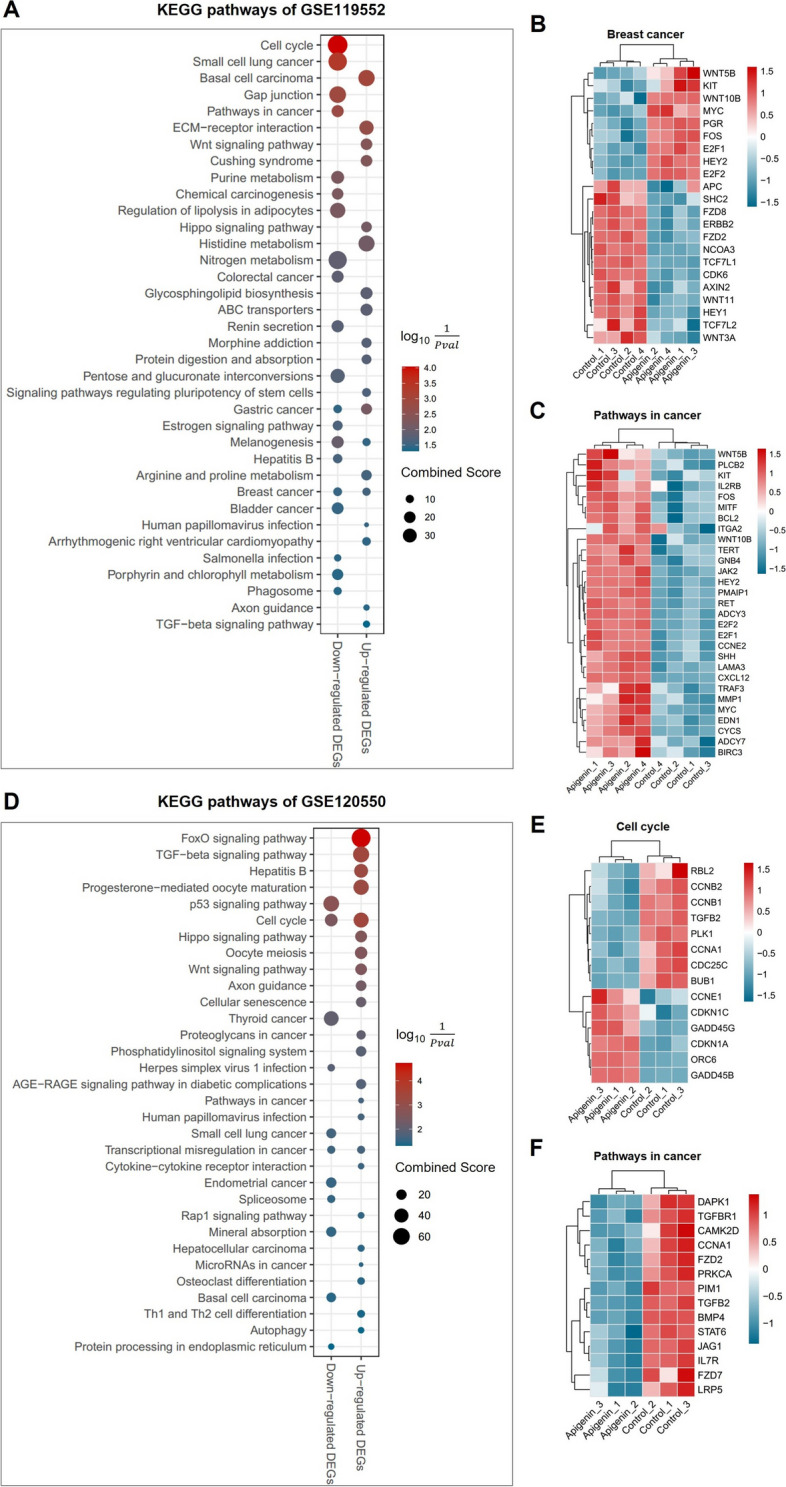
Validation of Apigenin treatment on human breast cancer cells using GEO datasets. **A**,** D** Scatter plots illustrating the significant KEGG pathways enriched by the up- and down-regulated differentially expressed genes (DEGs) within indicated GEO datasets. The *y*-axis represents the name of pathway, and the *x*-axis represents the up- and down-regulated DEGs. Dot size represents the combined score, and the colour indicates the log_10_($\frac{1}{Pvalue}$). (**B**, **C**, **E**, **F**) Heatmaps showing transcriptional levels of genes enriched in each representative pathway of Apigenin-treated breast cancer cells compared to vehicle

DEGs were screened from the obtained GEO datasets (Supplementary Fig. 5), and KEGG enrichment analysis was performed to identify enriched pathways. In MCF7 cell treated with Apigenin, both up- and down-regulated DEGs showed significant enrichment in the Breast cancer pathway (Fig. 9A, Supplementary Table 14), highlighting the crucial role of Apigenin in the treatment of breast cancer. Furthermore, the expression levels of genes associated with breast cancer were significantly altered by the treatment of Apigenin (Fig. 9B). In MDA-MB-231 cells, Cell cycle was commonly enriched from both up- and down-regulated DEGs upon Apigenin treatment (Fig. 9D, E, Supplementary Table 15). Additionally, we noticed that Pathways in cancer was significantly enriched in both Apigenin treated MCF7 and MDA-MB-231 cells, and genes involved in this pathway exhibited significant changes in expression levels (Fig. 9C, F), indicating the potential anti-cancer effects of Apigenin. These findings from the GEO dataset analysis further support the role of Apigenin in breast cancer treatment, especially its impact on breast cancer pathways, cell cycle regulation, and potential anti-cancer effects through modulation of genes involved in cancer-related pathways.

## Discussion

The anti-tumour activity of *L. sinense* has been previously discussed to be associated with its immunomodulatory activity [6]. In line with these findings, our current analysis emphasizes the strong association between the biological activity of *L. sinense* and cancer-related pathways, suggesting a potential role of *L. sinense* in cancer treatment. Through network pharmacology analysis, we identified several enriched pathways related to cancer regulation, with “Pathways in cancer” being the top ranked KEGG pathway. Additional cancer-related signalling pathways, including Chemical carcinogenesis, Prostate cancer, Proteoglycans in cancer and Central carbon metabolism in cancer, were also significantly enriched by the targets of *L. sinense*. These findings underscore the importance of *L. sinense* in cancer regulation. Moreover, the significant enrichment of the Cell cycle pathway in our analysis further supports our previous findings that the water extract of *L. sinense* leads to cell cycle arrest at the G2/M phase [13]. Additionally, the ethanol extract of *L. sinense* demonstrates a remarkable inhibitory effect on the growth of various breast cancer cell lines, suggesting the considerable potential of *L. sinense* as an adjuvant of therapeutic agent for the treatment of breast cancer.

This study has identified 15 natural components as the main active compounds in *L. sinense*, with the majority of them demonstrating potent anti-cancer properties. For instance, gallic acid (3,4,5-trihydroxybenzoic acid), a major phenolic acid commonly found in plants and fruits, exhibits a wide range of biological activities, including antioxidant, anti-microbial, anti-inflammatory and anti-cancer effects [41]. It was showed that gallic acid effectively against metastasis in various cancer cell types such as myeloid leukemia, breast cancer and ovarian cancer by modulating multiple signalling pathways, including Akt/mTOR, ERK, MMPs, NFκB, PTEN/Akt/HIF-1α/VEGF pathways [42–44]. Apigenin, a naturally occurring plant flavone, has gained recognition as a promising cancer chemopreventive agent. It exhibits remarkable antioxidant, anti-mutagenic, anti-inflammatory, antibacterial and antiviral effects [45–48]. Recent research has extensively investigated Apigenin for its anti-cancer activities, showing broad-spectrum effects across various cancer types, including colorectal cancer, breast cancer, liver cancer, lung cancer, melanoma, prostate cancer, and osteosarcoma [49–54]. Studies have revealed that Apigenin exerts its anti-cancer properties potentially by inducing cell cycle arrest, triggering apoptosis, inducing autophagy, inhibiting migration/invasion, attenuating drug resistance, or stimulating immune responses in various cancer types both in vitro and in vivo [45, 55]. Luteolin, another flavone compound found in various plants and medicinal herbs, exhibits diverse biological effects, such as antioxidant and anti-inflammatory properties. Luteolin has also demonstrated anti-cancer activity against multiple types of human cancers, including lung cancer, breast cancer, glioblastoma cancer, prostate cancer, colon cancer, and pancreatic cancer [56]. In the context of carcinogenesis, luteolin impedes cancer progression through various mechanisms, including the suppression of kinases, regulation of the cell cycle, induction of apoptotic cell death, and reduction of transcription factors, thereby inhibiting cell transformation, metastasis, invasion, and angiogenesis [57].

The hub network analysis revealed that Apigenin as the key component in *L. sinense* that can directly interact with 6 hub targets, including *AKT1*, *EGFR*, *SRC*, *ESR1*, *GSK3B* and *PTGS2*. By using molecular docking method, we observed that Apigenin binding with these hub targets tightly and forming various interaction bonds at the binding sites. We further checked the mRNA expressions of these 6 hub targets in the TCGA cohort using UCSCXenaShiny (*v1.1.9*) (A comprehensive description of UCSCXenaShiny methodology can be found in reference [58]). The results revealed significant up- or down-regulation of these hub targets in most of the 33 TCGA cancer types in mRNA levels (Supplementary Fig. 6A-F). In addition, these target genes also predicted overall survival in multiple cancer types (Supplementary Fig. 6G). These results suggesting the Apigenin in *L. sinense* may play a key role in regulating cancer process and disease-related signalling pathways.

*L. sinense* has traditionally been used to replenish blood in the body, and treat conditions such as haemostasis, anaemia, bleeding and menorrhagia in Chinese folk medicine [1]. However, there is no direct evidence to prove the blood-enriching function of *L. sinense* so far. Our network pharmacology and RNA-seq analyses yielded a noteworthy observation that the biological process “blood vessel development” was significantly enriched in both *L. sinense* targets and the DEGs identified in MDA-MB-468 breast cancer cells treated with LSE. These findings provide valuable novel insights into the mechanisms underlying *L. sinense* potential to boost blood vessels. It is well known that anaemia is a common diagnosis in patients with cancer that may affect both quality of life and survival [59]. Cancer can directly cause or exacerbate anaemia either by suppressing haematopoiesis, cytokine-induced iron sequestration, or reduced red blood cell production [60]. Treatment of anaemia can significantly improve patients’ quality of life and potentially enhance clinical outcomes. Therefore, the blood-enriching property of *L. sinense* may help alleviate cancer-related symptoms. Additionally, the immunomodulatory activity of *L. sinense* may also play a critical role in this process. Further exploration and verification using high-performance liquid chromatography (HPLC) and disease models are required to elucidate the mechanisms underlying these effects and to better understand the blood-enriching and anti-tumour activities of *L. sinense*.

## Conclusions

Our study predicts that the biological activities of *L. sinense* are strongly associated with breast cancer and multiple cancer-related pathways. The active compound Apigenin in *L. sinense* appears to have a crucial role against cancer. Experimental assessments of the ethanol extract of *L. sinense* have demonstrated a notable growth inhibitory effect on multiple breast cancer cell lines. Additionally, RNA-seq analysis unveiled global transcriptomic alterations in breast cancer cells treated with LSE potentially linked to pathways associated with breast cancer and hematopoietic cell lineage. GEO datasets validation indicates the involvement of breast cancer pathway upon treatment with Apigenin in human breast cancer cells. These findings highlight the potential of *L. sinense* as a promising therapeutic agent for the treatment of breast cancer. Further research and clinical investigations are warranted to explore the full therapeutic potential of *L. sinense* in combating breast cancer.

### Supplementary Information

**Additional file  1.**

**Additional file  2.**

**Additional file 3.**

**Additional file 4.**

**Additional file  5.**

**Additional file 6.**

**Additional file  7.**

**Additional file 8.**

**Additional file 9.**

**Additional file 10.**

**Additional file  11.**

**Additional file  12.**

**Additional file  13.**

**Additional file  14.**

**Additional file 15.**

**Additional file  16.**

**Additional file  17.**

**Additional file 18.**

**Additional file  19.**

**Additional file  20.**

**Additional file  21.**

**Additional file  22.**

**Additional file 23.**

**Additional file  24.**

**Additional file  25.**

## Data Availability

All data generated or analysed in this study are included in the article and its supplementary information files. The RNA-Seq data have been deposited in the Gene Expression Omnibus (GEO) database (accession code GSE244469).

## Abbreviations

2D: Two dimensional
3D: Three dimensional
GEO: Gene Expression Omnibus
GO: Gene Ontology
KEGG: Kyoto Encyclopedia of Genes and Genomes
*L. sinense*: *Limonium Sinense* (Girard) Kuntze
LSE: *L. sinense* ethanol extract
RNA-seq: RNA-sequencing
