# Supplementary material for: Integrated network pharmacology and cellular assay reveal the biological mechanisms of Limonium sinense (Girard) Kuntze against breast cancer
Source: BMC Complement Med Ther. Author manuscript; Available in PMC 2023 Nov 21. (PMC10644419; doi:10.1186/s12906-023-04233-z)

# ADDITIONAL FILE

# Integrated network pharmacology and cellular assay reveal the biological mechanisms of *Limonium sinense* (Girard) Kuntze against breast cancer

Hualong Zhao[1,2], Siyuan Wang[2], Philip T.F. Williamson[2,3], Rob M. Ewing[2,3], Xinhui Tang[1], Jialian Wang[1*], Yihua Wang[2,3**]

[1]School of Marine and Biological Engineering, Yancheng Teachers' University, Xiwang Road, Yancheng 224002, PR China.

[2]Biological Sciences, Faculty of Environmental and Life Sciences, University of Southampton SO17 1BJ, UK.

[3]Institute for Life Sciences, University of Southampton, Southampton SO17 1BJ, UK.

[*]Corresponding author. School of Marine and Biological Engineering, Yancheng Teachers' University, Xiwang Road, Yancheng 224002, PR China

[**]Corresponding author. Biological Sciences, Faculty of Environmental and Life Sciences, University of Southampton SO17 1BJ, UK

# Table of contents


*Supplementary Methods* ........ *4*

**1. GSEApy protocol in python** ........ 4

**2. R Scripts** ........ 6

**2.1. R codes for** Figure 5C ........ 6

**2.2. R codes for obtaining rawdata of LSE-treated MDA-MB-468 cells gene dataset** ........ 7

**2.3. R codes for** Figure 8A ........ 8

**2.4. R codes for** Figure 8B ........ 9

**2.5. R codes for** Figure 8C ........ 9

**2.6. R codes for** Figure 8D, E ........ 10

**2.7. R codes for obtaining rawdata of GEO datasets** ........ 11

**2.8. R codes for** Figure 9A and 9D ........ 12

**2.9. R codes for** Figure 9B, 9C, 9E and 9F ........ 13

**2.10. R codes for Supplementary** Figure 4A, 4B ........ 14

*Supplementary Figures* ........ *15*

**Supplementary Figure 1. Workflow for compound screening of *L. sinense*** ........ 15

**Supplementary Figure 2. ClueGo Biological Process analysis of the *L. sinense*** ........ 16

**Supplementary Figure 3. Target-Pathway (TP) network of *L. sinense*** ........ 17

**Supplementary Figure 4. Principal Component Analysis (PCA) of RNA-seq based expression data from *L. sinense*** ........ 18

**Supplementary Figure 5. Global transcriptomic changes in human breast cancer cells exposed to Apigenin.** ........ 18

**Supplementary Figure 6. Relative mRNA expression and Overall Survival of the 6 hub target genes in TCGA cancers.** ........ 19

*Supplementary Tables* ........ *20*

**Supplementary Table 1. Identified Compounds in *L. sinense*** ........ 20

**Supplementary Table 2. Potential targets of *L. sinense*** ........ 20

**Supplementary Table 3. Details of the information of Compound-Target network** ........ 20

**Supplementary Table 4. Details of the core targets identified from PPI network** ........ 20

**Supplementary Table 5. Details of the KEGG pathways enriched from *L. sinense* targets** ........ 20

**Supplementary Table 6. Details of the Biological Process terms enriched by *L. sinense* targets** ........ 20

**Supplementary Table 7. Hub network information identified using Cytohubba** 20

**Supplementary Table 8. Details of DisGeNET analysis** 20

**Supplementary Table 9. DEGs identified in LSE-treated MDA-MB-468 cells** 20

**Supplementary Table 10. GSEA hallmark analysis from LSE-treated MDA-MB-468 cells** 20

**Supplementary Table 11. KEGG pathways enriched from LSE-treated MDA-MB-468 cells** 20

**Supplementary Table 12. BP terms enriched from LSE-treated MDA-MB-468 cells** 20

**Supplementary Table 13. Details of the GEO datasets collected for Apigenin treatment** 20

**Supplementary Table 14. Details of the KEGG enrichment analysis (GSE119552)** 20

**Supplementary Table 15. Details of the KEGG enrichment analysis (GSE120550)** 20

**Supplementary Table 16. Gene list for enrichment analysis** 20

**Raw_data_Table 1. GSE119552_DEGs_from_GEO2** 20

**Raw_data_Table 2. GSE120550_DEGs_from_GEO2** 20

**Raw_data_Table 3. GSE119552_breast_cancer_genes** 20

**Raw_data_Table 4. GSE119552_pathways_in_cancer_genes** 20

**Raw_data_Table 5. GSE120550_cell_cycle_genes** 20

**Raw_data_Table 6. GSE120550_ pathways_in_cancer_genes** 20

**Raw_data_Table 7. Cell viability assay of breast cancer cell lines treatment with LSE** 20

**Raw_data_Table 8. Cell viability of MDA-MB-468 cells treatment with LSE in 3D culture** 20

***Raw data for 3D culture*** 21

***Raw data for 2D culture*** 22

# Supplementary Methods

## 1. GSEApy protocol in python

The GSEApy analysis was performed in Colab Notebooks under python environment.

Upload the gene list (Supplementary Table 16) into Colab Notebooks as default path.

#Installation

```
%pip install gseapy

%load_ext autoreload
%autoreload 2
import pandas as pd
import gseapy as gp
import matplotlib.pyplot as plt
from gseapy import Biomart
bm = Biomart()
import numpy as np
import pandas as pd
from gseapy import barplot, dotplot
from matplotlib import figure
```

#Run GSEApy

```
gene_list=pd.read_csv("/content/
Gene_list_for_enrichment_analysis.txt",header=None,sep="\t")
```

#KEGG enrichment

```
enr_KEGG = gp.enrichr(gene_list= gene_list,
                 gene_sets=['KEGG_2021_Human'],
                 organism='human',
                 outdir=None,
                 )

enr_KEGGFrame=pd.DataFrame(data=enr_KEGG.results,columns=['Gene_s
et','Term','Overlap','P-value','Adjusted P-value','Odds
Ratio','Combined Score','Genes'])

enr_KEGGFrame.to_csv('enr_KEGG_data',index=False)
ax_KEGG = dotplot(enr_KEGG.res2d, title='KEGG pathway',
top_term=20,cmap='viridis_r',size=10, figsize=(4, 8))
```

#GO term enrichment

```
enr_GO = gp.enrichr(gene_list=gene_list,
                 gene_sets=['GO_Biological_Process_2021','GO_Mole
cular_Function_2021','GO_Cellular_Component_2021'],
                 organism='human',
                 outdir=None,
                 )

enr_GOFrame=pd.DataFrame(data=enr_GO.results,columns=['Gene_set',
'Term','Overlap','P-value','Adjusted P-value','Odds
Ratio','Combined Score','Genes'])

enr_GOFrame.to_csv('enr_GO_data',index=False)
```

```
ax_GO = dotplot(enr_GO.results,
             column="Adjusted P-value",
             x='Gene_set',
             size=6,
             top_term=10,
             figsize=(3,10),
             title="GO Terms",
             xticklabels_rot=45,
             show_ring=False,
             )
```

## 2. R Scripts

Raw data were imported into RStudio (version 4.2.0), Microsoft Windows (version 11) and R scripts were run.
# Set the working directory before run
# setwd("C:/Users/hz2u19/Downloads")

### 2.1. R codes for Figure 5C

```
BiocManager::install("disgenet2r")
library(disgenet2r)

hub_gene_dis<- gene2disease(gene = c( "AKT1", "EGFR", "SRC", "ESR1",
                                      "GSK3B","PTGS2"), database = "ALL")
hub_results<- extract(hub_gene_dis)
res_enrich<- disease_enrichment(entities = c("AKT1","EGFR","SRC","ESR1",
                                             "GSK3B","PTGS2"))
table1<- res_enrich@qresult[, c("Description", "FDR","Ratio","BgRatio")]
write.csv(table1, “hub_genes_DisGeNET_data.csv”)
plot(res_enrich,class = "Enrichment", count = 3, cutoff = 0.05, nchars =50)
```

**2.2. R codes for obtaining rawdata of LSE-treated MDA-MB-468 cells gene dataset**

```
library(DESeq2)

#Download the rawdata from GSE244469
#  https://www.ncbi.nlm.nih.gov/geo/query/acc.cgi?acc=GSE244469

#Prepare the row annotation from
#https://github.com/theislab/scvelo_notebooks/blob/master/data/biomart/mart_export
#_human.txt.

lse_counts<- read.csv("lse_rawcount.csv", header = TRUE)
annotation<- read.table("mart_export_human.txt", header = T, sep = "\t")
colnames(annotation) <- c("Geneid", "Gene_name")
expr<- merge(lse_counts, annotation, by = "Geneid")
expr<- expr[, c(8, 2:7)]
expr <- aggregate(x=expr, by=list(expr$Gene_name), FUN=median)
rownames(expr)<- expr$Group.1
expr<- expr[, -1]
write.csv(expr, "LSE_rawdata.csv")

lse_counts<- read.csv("LSE_rawdata.csv", row.names = 1)
lse_counts<- na.omit(lse_counts)
condition<- factor(c("Control", "Control","Control","LSE","LSE","LSE"))
coldata<- data.frame(row.names = colnames(lse_counts), condition)
dds<- DESeqDataSetFromMatrix(countData = round(lse_counts), colData = coldata,
                              design = ~condition)
dds<- DESeq(dds)
dds<- estimateSizeFactors(dds)
se<- SummarizedExperiment(log2(counts(dds, normalized = TRUE) +1),
                           colData = colData(dds))
plotPCA(DESeqTransform(se))
res<- results(dds, contrast = c("condition", "LSE", "Control"))
res<- as.data.frame(res)
raw_count<- counts(dds, normalized = FALSE)
raw_count<- as.data.frame(raw_count)
normalizedCounts2<- counts(dds, normalized = TRUE)
res<- data.frame(res, normalizedCounts2)
res<- na.omit(res)
write.csv(res, file = "LSE_DESeq_data.csv")
```

**2.3. R codes for** Figure 8A

```
library(ggplot2)
library(RColorBrewer)
library(ggrepel)
library(tidyverse)

volcano_lse<- read.csv("LSE_DESeq_data.csv", row.names = 1)
volcano_lse <- volcano_lse %>%
  mutate(gene_type = case_when(log2FoldChange >= 2 & padj <= 0.05 ~ "Up-
regulated",
                               log2FoldChange <= -2 & padj <= 0.05 ~ "Down-
regulated",
                               TRUE ~ "Not significant"))

volcano_lse %>%
  count(gene_type)

cols <- c("Up-regulated" = "#bb0c00", "Down-regulated" = "#00AFBB", "Not
significant" = "grey")
sizes <- c("Up-regulated" = 1, "Down-regulated" = 1, "Not significant" = 1)
alphas <- c("Up-regulated" = 1, "Down-regulated" = 1, "Not significant" = 0.5)

ggplot(data = volcano_lse, aes(x = log2FoldChange, y = -log10(padj))) +
  geom_vline(xintercept = c(-2, 2), col = "gray", linetype = 'dashed') +
  geom_hline(yintercept = -log10(0.05), col = "gray", linetype = 'dashed') +
  geom_point(aes(colour = gene_type)) +
  scale_colour_manual(values = cols) +
  scale_size_manual(values = sizes) +
  scale_alpha_manual(values = alphas) +
  scale_x_continuous(breaks = c(seq(-10, 10, 2)),
                     limits = c(-12, 12)) +
  labs(title = "Gene expression changes of Control vs LSE treatment",
       x = "Log2FoldChange", y = "-Log10(Padj)", colour = "Expression") +
  theme_bw() +
  theme(panel.border = element_rect(colour = "black", fill = NA, size= 0.5),
        panel.grid.minor = element_blank(),
        panel.grid.major = element_blank()) +
  theme(legend.position = c(.98, .90), legend.justification = c("right", "top"),
    legend.box.just = "right", legend.margin = margin(6, 6, 6, 6))

write.csv(volcano_lse, "lse_degs.csv")
```

**2.4. R codes for** Figure 8B

```
library(pheatmap)
library(RColorBrewer)

lse_deg_heatmap<- read.csv("LSE_DEGs.csv", row.names = 1)
lse_deg_heatmap<- lse_deg_heatmap[,c(7:12)]
pheatmap(lse_deg_heatmap,
         scale = "row",
         treeheight_row = 40,
         treeheight_col = 40,
         annotation_row = NA,
         annotation_names_row = FALSE,
         cluster_rows = T,
         cluster_cols = T,
         show_rownames = F,
         color = colorRampPalette(c("deepskyblue4","white", "red3"))(500),
         border_color = "grey90",
         angle_col = 45,
         fontsize_col = 10)
```

**2.5. R codes for** Figure 8C

```
library(RColorBrewer)
library(ggplot2)

lse_hallmark<- read.csv("lse_hallmark_analysis.csv", header = T)
ggplot(lse_hallmark, aes(x = NES, y = reorder(NAME, -FDR),
                         color = -log10(FDR),
                         size = SIZE))+
  geom_point()+
  scale_color_gradient(low = "deepskyblue4", high = "red3")+
  theme(plot.subtitle = element_text(size=11, angle=0))+
  theme(axis.title.y = element_text(size=11, angle=0)) +
  theme(axis.title.x = element_text(size=11, angle=0)) +
  theme(legend.position="bottom") +
  theme_bw()+
  theme(panel.grid.major = element_line(color = "grey80",
                                        size = 0.05, linetype = 1)) +
  labs(x="NES", y="") +
  ggtitle("GSEA Hallmark") +
  theme(plot.title = element_text(hjust = 0.5))
```

**2.6. R codes for** Figure 8D, E

```
library(RColorBrewer)
library(ggplot2)
library(gridExtra)
library(dplyr)

lse_up_degs_kegg<- read.csv("lse_up_degs_kegg.csv", header = T)
ggplot(lse_up_degs_kegg, aes(x = Gene_set, y = reorder(Term, log10.1.P.value.),
                                         color = log10.1.P.value., size = X.Genes.in.set))+
  geom_point()+
  scale_color_gradient(low = "dodgerblue3", high = "brown1")+
  theme_bw()+
  labs(x="", y="", title="")+
  theme(axis.title.x = element_blank(),
        axis.ticks = element_blank()) +
  theme(axis.text.x = element_text(angle = 270, hjust = 0.1, vjust = 0.5))


lse_down_degs_kegg<- read.csv("lse_down_degs_kegg.csv", header = T)
ggplot(lse_down_degs_kegg, aes(x = Gene_set, y = reorder(Term, log10.1.P.value.),
                                         color = log10.1.P.value., size = X.Genes.in.set))+
  geom_point()+
  scale_color_gradient(low = "dodgerblue3", high = "brown1")+
  theme_bw()+
  labs(x="", y="", title="")+
  #theme(axis.text.x = element_text(angle = 270, hjust = 0.1, vjust = 0.5)) +
  theme(axis.title.x = element_blank(),
        axis.ticks = element_blank())
```

## 2.7. R codes for obtaining rawdata of GEO datasets

```
library(GEOquery)
library(limma)
```

**#GSE119552**

```
gse119552 <- getGEO("GSE119552", GSEMatrix =TRUE, AnnotGPL=FALSE)
if (length(gse119552) > 1) idx <- grep("GPL16699", attr(gse119552, "names")) else
idx <- 1
gse119552 <- gse119552[[idx]]
gse119552_rawdata<- cbind(fData(gse119552), exprs(gse119552))
gse119552_rawdata <-
aggregate(x=gse119552_rawdata[,2:(ncol(gse119552_rawdata))],
by=list(gse119552_rawdata $Gene_Symbol), FUN = median)
gse119552_rawdata <- na.omit(gse119552_rawdata)
rownames(gse119552_rawdata)<- gse119552_rawdata $Group.1
gse119552_rawdata <- gse119552_rawdata [,-1]
write.csv(gse119552_rawdata, "GSE119552_rawdata.csv")
```

**#GSE120550**

```
gse120550 <- getGEO("GSE120550", GSEMatrix =TRUE, AnnotGPL=FALSE)
if (length(gse120550) > 1) idx <- grep("GPL17692", attr(gse119552, "names")) else
idx <- 1
gse120550 <- gse120550[[idx]]
gse120550_rawdata<- cbind(fData(gse120550), exprs(gse120550))
gse120550_rawdata <-
aggregate(x=gse120550_rawdata[,2:(ncol(gse120550_rawdata))],
by=list(gse120550_rawdata $Gene_Symbol), FUN = median)
gse120550_rawdata <- na.omit(gse120550_rawdata)
rownames(gse120550_rawdata)<- gse120550_rawdata $Group.1
gse120550_rawdata <- gse120550_rawdata [,-1]
write.csv(gse120550_rawdata, "GSE120550_rawdata.csv")
```

**2.8. R codes for** Figure 9A and 9D

```
library(ggplot2)
library(RColorBrewer)
library(gridExtra)
library(dplyr)

gse119552_kegg<- read.csv("gse119552_kegg_genes.csv", header = T)
ggplot(gse119552_kegg, aes(x = DEGs, y = reorder(Term, -P.value),
                            color = log10(1/P.value), size = Combined.Score))+
   geom_point()+
   scale_color_gradient(low = "deepskyblue4", high = "red3")+
   theme_bw()+
   labs(x="", y="", title="")+
   theme(axis.text.x = element_text(angle = 270, hjust = 0.1, vjust = 0.5))+
   facet_grid(cols = vars(), scales = "free_x", space = "free_x")


gse120550_kegg<- read.csv("gse120550_kegg_genes.csv", header = T)
ggplot(gse120550_kegg, aes(x = DEGs, y = reorder(Term, -P.value),
                                     color = log10(1/P.value), size = Combined.Score))+
   geom_point()+
   scale_color_gradient(low = "deepskyblue4", high = "red3")+
   theme_bw()+
   labs(x="", y="", title="")+
   theme(axis.text.x = element_text(angle = 270, hjust = 0.1, vjust = 0.5))+
   facet_grid(cols = vars(), scales = "free_x", space = "free_x")
```

**2.9. R codes for** Figure 9B, 9C, 9E and 9F

```
library(pheatmap)

gse119552_breast_genes<- read.csv("gse119552_breastcancer_genes.csv",
row.names = 1)
pheatmap(gse119552_breast_genes, scale = 'row', treeheight_row = 20,
        treeheight_col = 20, annotation_row = NA,
        annotation_names_row = FALSE, cluster_rows = T, cluster_cols = T,
        color = colorRampPalette(c("deepskyblue4","white", "red3"))(500),
        border_color = "grey90", angle_col = 45, fontsize_col = 10)


gse119552_pic_genes<- read.csv("gse119552_pathwaysincancer_genes.csv",
row.names = 1)
pheatmap(gse119552_pic_genes, scale = 'row', treeheight_row = 20,
        treeheight_col = 20, annotation_row = NA,
        annotation_names_row = FALSE, cluster_rows = T, cluster_cols = T,
        color = colorRampPalette(c("grey70","white", "deepskyblue4"))(500),
        border_color = "grey90", angle_col = 45, fontsize_col = 10)


gse120550_cellcycle_genes<- read.csv("gse120550_cellcycle_genes.csv", row.names
= 1)
pheatmap(gse120550_cellcycle_genes, scale = 'row', treeheight_row = 20,
        treeheight_col = 20, annotation_row = NA,
        annotation_names_row = FALSE, cluster_rows = T, cluster_cols = T,
        color = colorRampPalette(c("deepskyblue4","white", "red3"))(500),
        border_color = "grey90", angle_col = 45, fontsize_col = 10)


gse120550_pic_genes<- read.csv("gse120550_pathwaysincancer_genes.csv",
row.names = 1)

pheatmap(gse120550_pic_genes, scale = 'row', treeheight_row = 20,
        treeheight_col = 20, annotation_row = NA,
        annotation_names_row = FALSE,
        cluster_rows = T, cluster_cols = T,
        color = colorRampPalette(c("grey70","white", "red3"))(500),
        border_color = "grey90",
        angle_col = 45, fontsize_col = 10)
```

**2.10. R codes for Supplementary** Figure 4A, 4B

```
library(EnhancedVolcano)

gse119552_vol<- read.csv("GSE119552_DEGs_from_GEO2.csv", header = T)
EnhancedVolcano(gse119552_vol, lab = NA,
                selectLab = NULL,
                subtitle = "DEGs of GSE119552",
                subtitleLabSize = 18,
                x = 'logFC', y = 'P.Value',
                ylim = c(0, 12),
                pCutoff = 0.05,
                FCcutoff = 1,
                title = "")


gse120550_vol<- read.csv("GSE120550_DEGs_from_GEO2.csv", header = T)
EnhancedVolcano(gse120550_vol, lab = NA, selectLab = NULL,
                subtitle = "DEGs of GSE120550",
                subtitleLabSize = 18,
                x = 'logFC', y = 'P.Value',
                ylim = c(0, 10), xlim = c(-4, 3),
                pCutoff = 0.05,
                FCcutoff = 1, title = "")
```

# Supplementary Figures

**Supplementary Figure 1**. Workflow for compound screening of *L. sinense*.

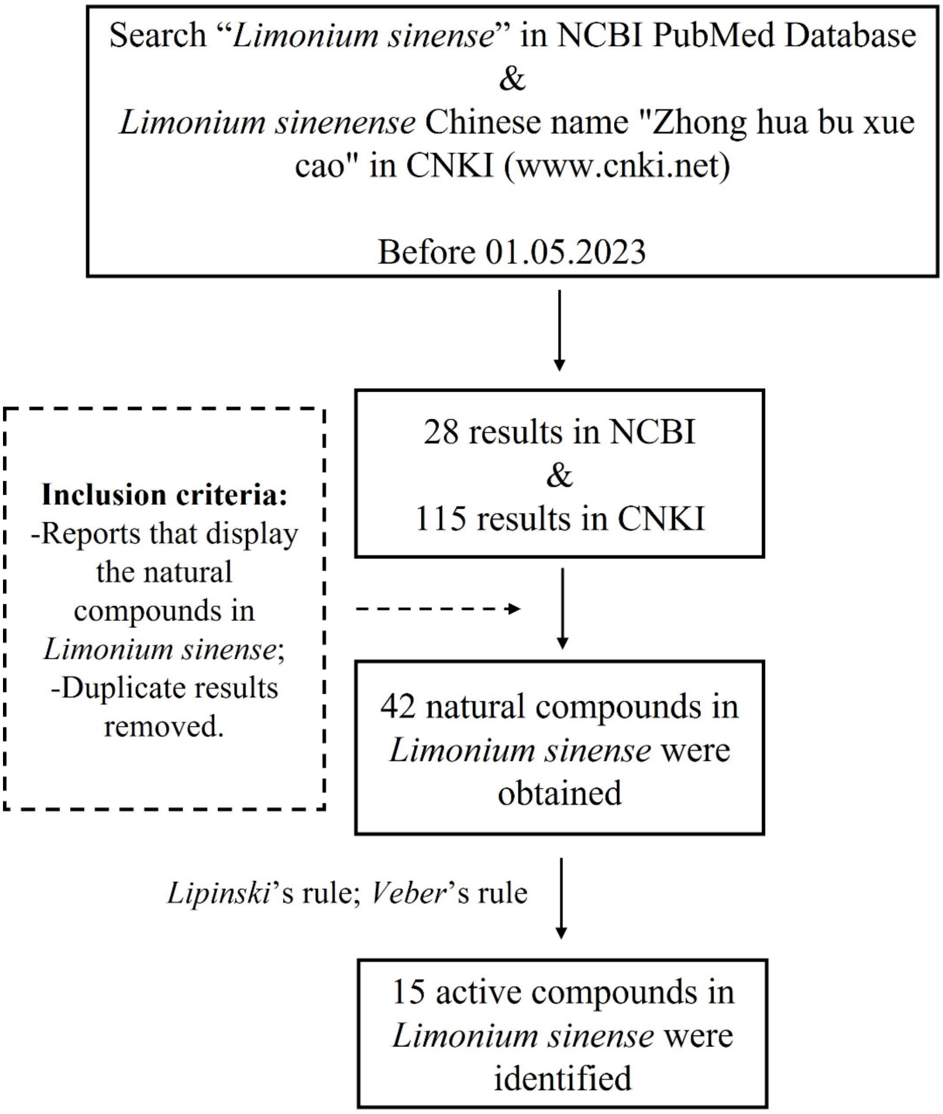

**Supplementary Figure 2**. **ClueGo Biological Process analysis of the *L. sinense* potential targets.** The network showing the individual terms within the "blood vessel enrichment" cluster from the enriched Biological Process. Biological Process terms are represented as nodes, and the node size represents the term enrichment significance. Functionally grouped networks are linked to their biological function, where only the significant term ($P \leq 0.05$) in the group is labelled.

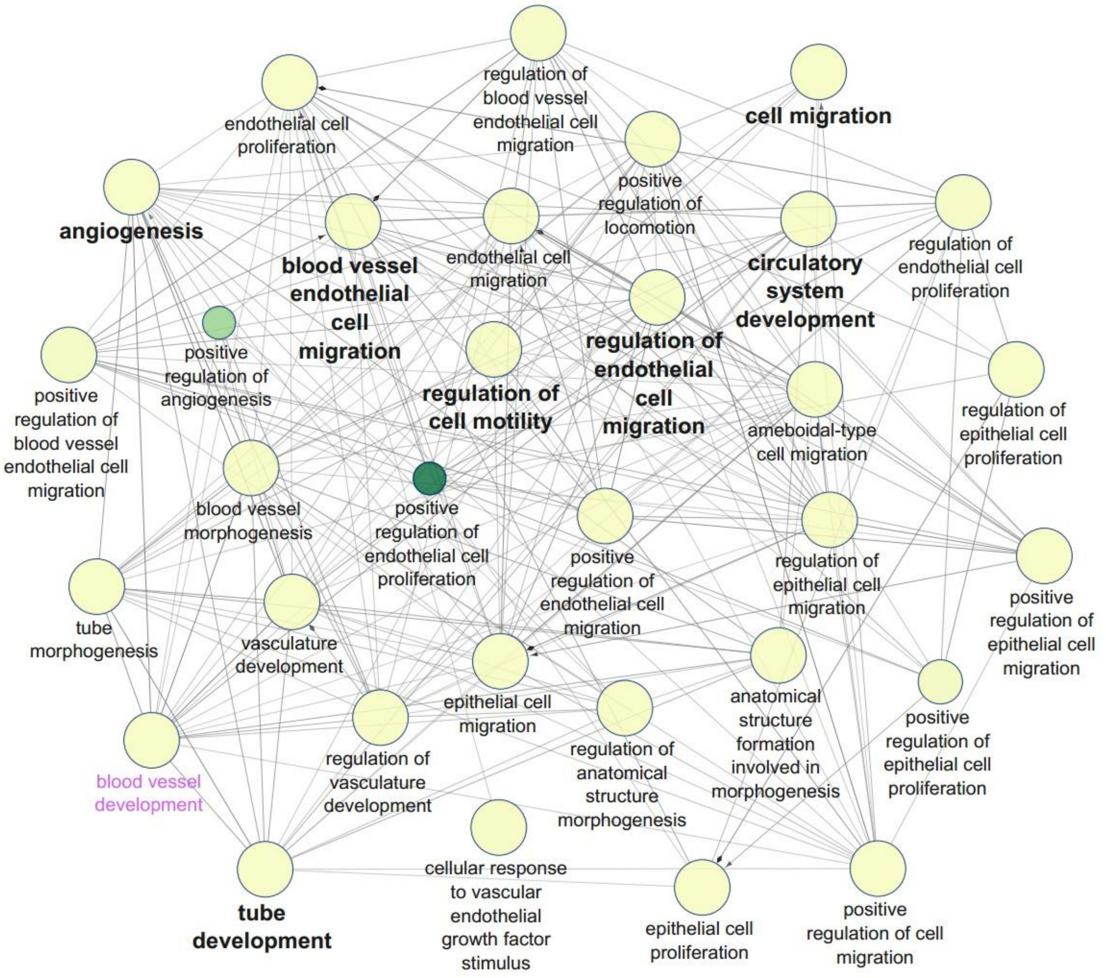

**Supplementary Figure 3**. **Target-Pathway (TP) network of *L. sinense*.** Graph consists of 20 signalling pathways and 194 pathway-related targets. The red V nodes represent the top 20 enriched KEGG pathways by the potential targets of *L. sinense*. The blue circles represent the relevant targets. The edges represent the relationship between signalling pathways and target nodes. The node size is proportional to the node degree in the network, and the width and colour of the edges is proportional to the edge betweenness centrality. The network is generated using the Cytoscape tool.

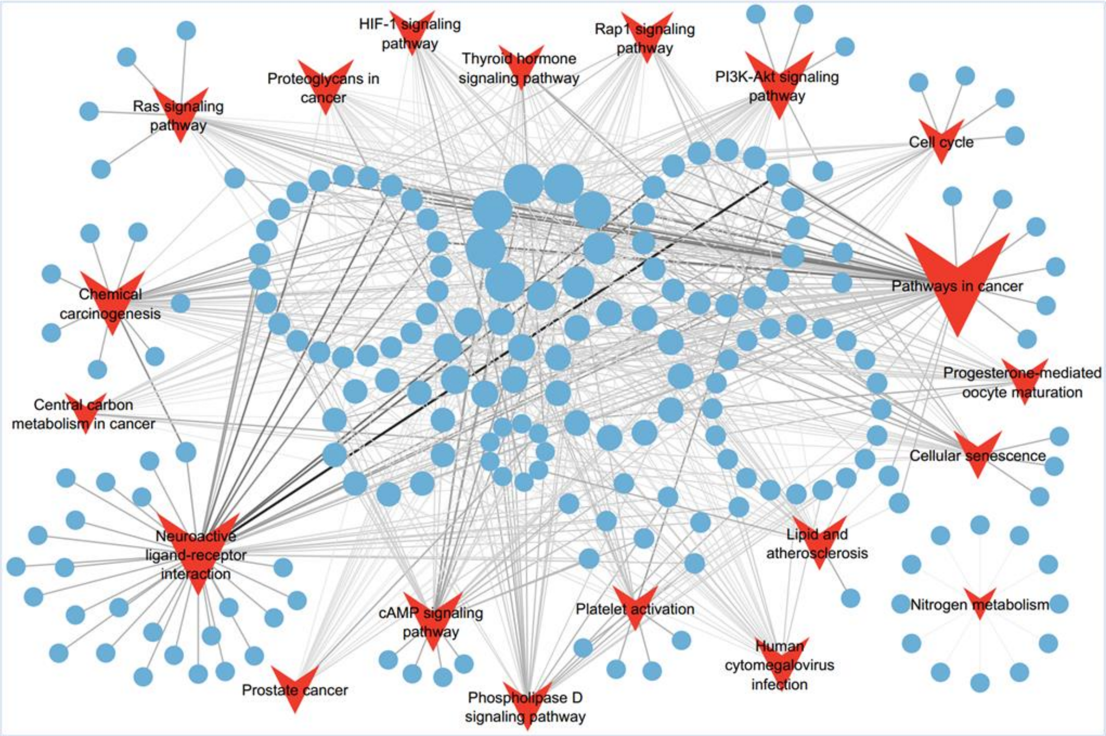

**Supplementary Figure 4**. **Principal Component Analysis (PCA) of RNA-seq based expression data from *L. sinense* ethanol extract (LSE)-treated MDA-MB-468 cells.**

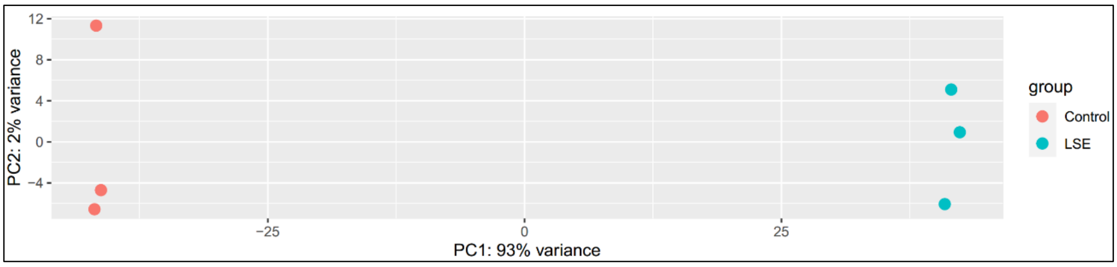


**Supplementary Figure 5. Global transcriptomic changes in human breast cancer cells exposed to Apigenin.** (**A** and **B**) Volcano plots showing up and down-regulated genes in Apigenin-treated human breast cancer cells within indicated GEO datasets. Log2FoldChange in *x*-axis and -Log10(*P*) in *y*-axis. Genes with different colours represent indicated changes in the figure legends.

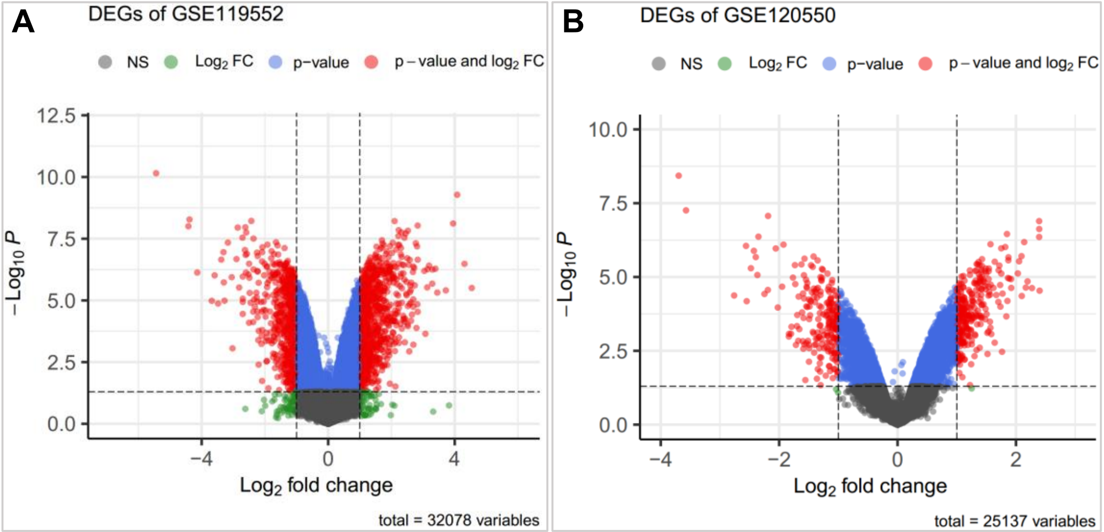

**Supplementary Figure 6**. **Relative mRNA expression and Overall Survival of the 6 hub target genes in TCGA cancers.** (**A-F**) Graphs showing the relative mRNA expression of each target gene obtained from the hub network in 33 TCGA cancers. ns, not significant, **P* < 0.05, ***P* < 0.01, ****P* < 0.001, *****P* < 0.0001. (**G**) Heatmap showing the Overall Survival of each target gene obtained from the hub network in 33 TCGA cancers. Red colour represents significant correlated with overall survival, while white colour represents not significant correlated with overall survival.

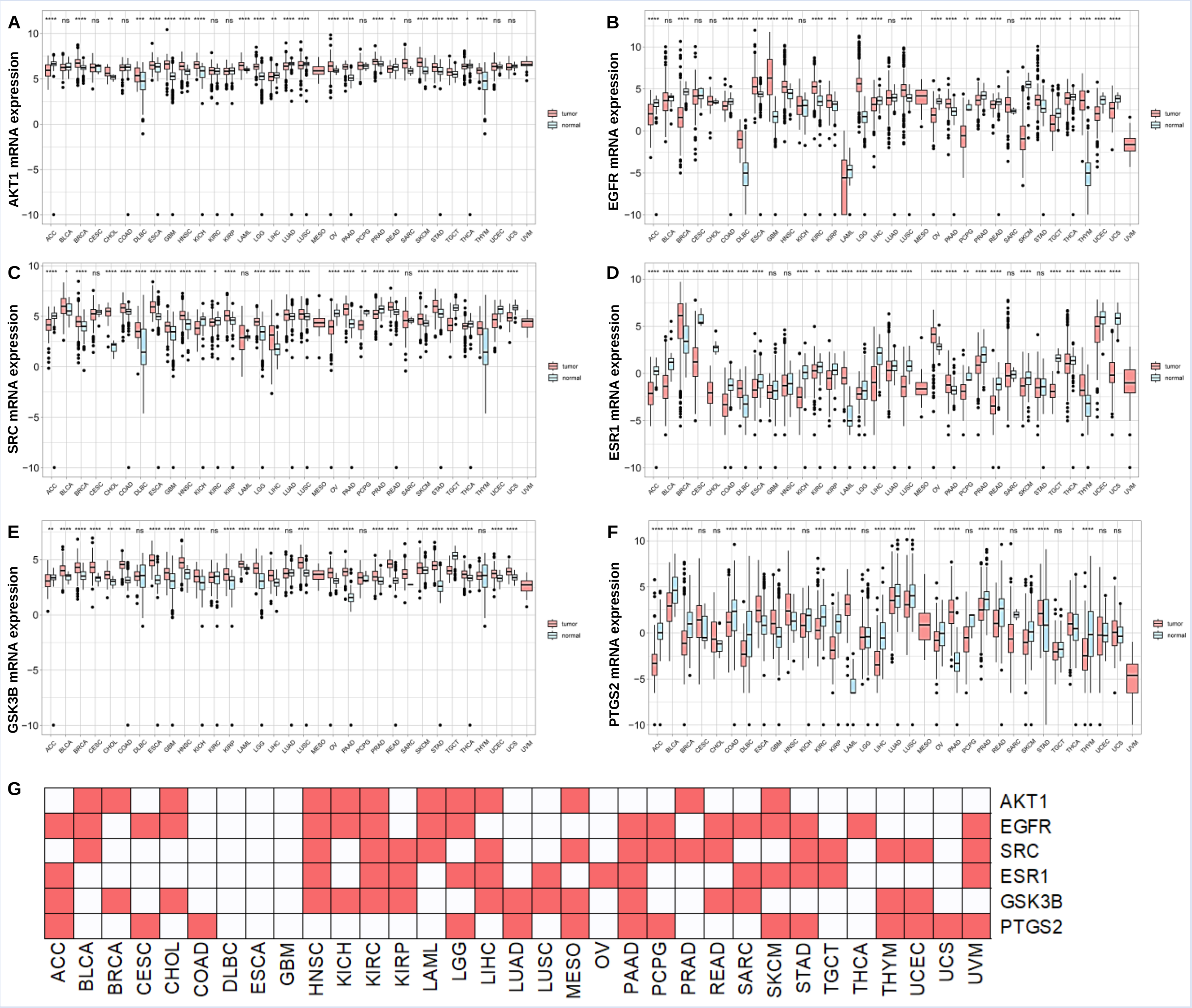

## Supplementary Tables

**Supplementary Table 1.** Identified Compounds in *L. sinense*

**Supplementary Table 2.** Potential targets of *L. sinense*

**Supplementary Table 3.** Details of the information of Compound-Target network

**Supplementary Table 4.** Details of the core targets identified from PPI network

**Supplementary Table 5.** Details of the KEGG pathways enriched from *L. sinense* targets

**Supplementary Table 6.** Details of the Biological Process terms enriched by *L. sinense* targets

**Supplementary Table 7.** Hub network information identified using Cytohubba

**Supplementary Table 8.** Details of DisGeNET analysis

**Supplementary Table 9.** DEGs identified in LSE-treated MDA-MB-468 cells

**Supplementary Table 10.** LSE hallmark analysis

**Supplementary Table 11.** KEGG pathways enriched from LSE-treated MDA-MB-468 cells

**Supplementary Table 12.** BP terms enriched from LSE-treated MDA-MB-468 cells

**Supplementary Table 13.** Details of the GEO datasets collected for Apigenin treatment

**Supplementary Table 14.** Details of the KEGG enrichment analysis (GSE119552)

**Supplementary Table 15.** Details of the KEGG enrichment analysis (GSE120550)

**Supplementary Table 16.** Gene list for enrichment analysis

**Rawdata Table 1.** GSE119552_DEGs_from_GEO2

**Rawdata Table 2.** GSE120550_DEGs_from_GEO2

**Rawdata Table 3.** GSE119552_breast_cancer_genes

**Rawdata Table 4.** GSE119552_pathways_in_cancer_genes

**Rawdata Table 5.** GSE120550_cell_cycle_genes

**Rawdata Table 6.** GSE120550_ pathways_in_cancer_genes

**Rawdata Table 7.** Cell viability assay of breast cancer cell lines treatment with LSE

**Rawdata Table 8.** Cell viability of MDA-MB-468 cells treatment with LSE in 3D culture

# Raw data for 3D culture

Control

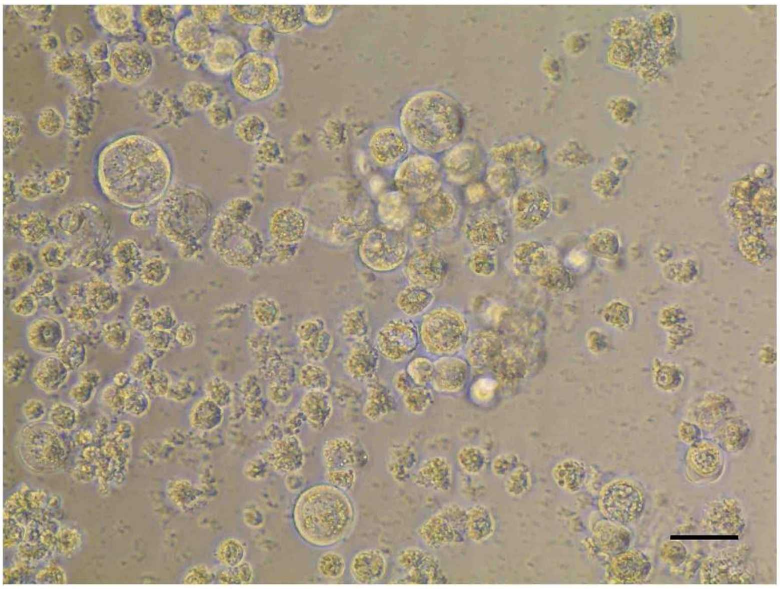

LSE-treated

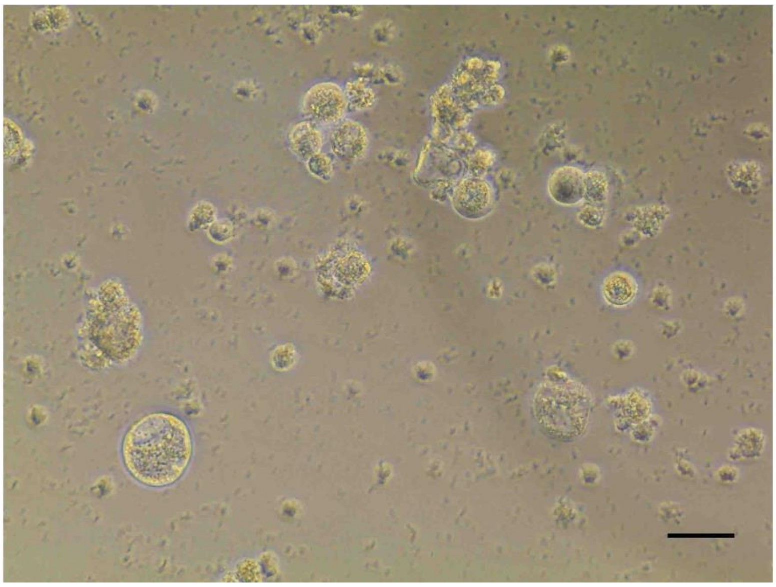

# Raw data for 2D culture

Control

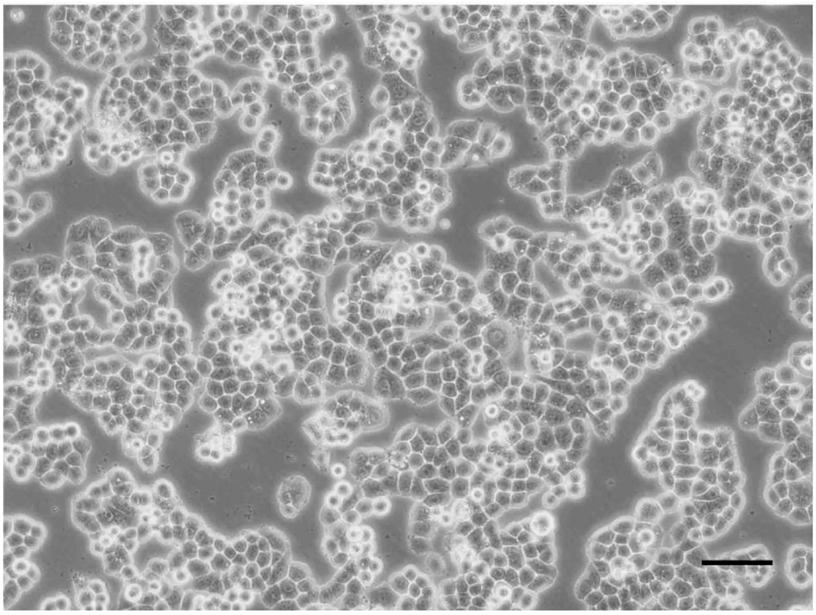

LSE-treated

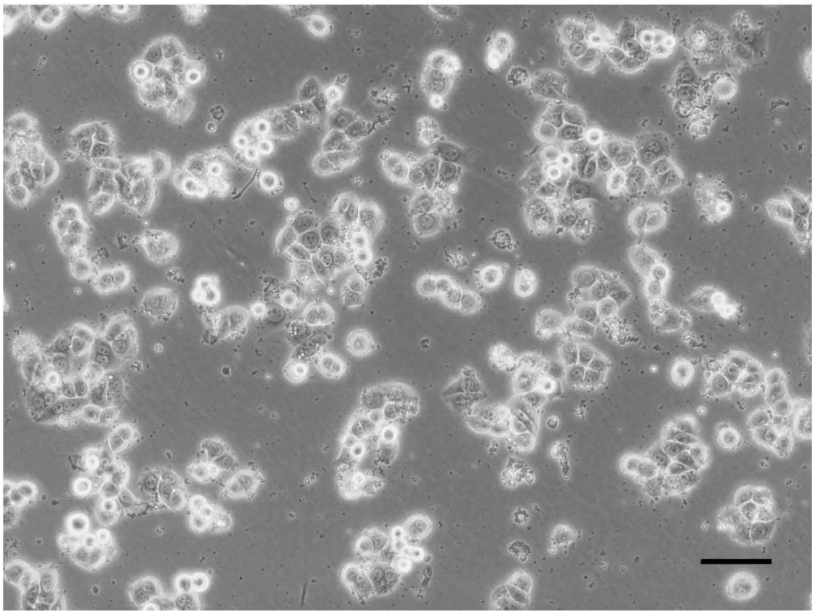

Supplement: Supplementary Materials [file EMS189939-supplement-Supplementary_Materials.pdf]
