# Supplementary material for: Integrated network pharmacology and cellular assay reveal the biological mechanisms of Limonium sinense (Girard) Kuntze against breast cancer
Source: BMC Complement Med Ther. Author manuscript; Available in PMC 2023 Nov 21. (PMC10644419; doi:10.1186/s12906-023-04233-z)
Supplement: Table S1 [file EMS189939-supplement-Table_S1.pdf]

Supplementary Table 1: Identified compounds in *L. sinense*

Compound Name	Compound Type	Extract Part	Reference
myricetin	Flavone	Leaves, Roots	[1], [2]
myricetin-3-O-α-rhamnopyranoside	Flavonol glycoside	Leaves, Roots	[1], [2]
Myricetin 3-O-β-galactopyranoside	Flavonol glycoside	Leaves	[1]
Myricetin 3-O-β-arabinopyranoside	Flavonol glycoside	Leaves	[1]
Myricetin 3-O-(2″-O-p-hydroxybenzoyl)-α-rhamnopyranoside	Flavonol glycoside	Leaves	[1]
Myricetin 3-O-(2″-O-galloyl)-α-rhamnopyranoside	Flavonol glycoside gallates	Leaves	[1]
Myricetin 3-O-(3″-O-galloyl)-α-rhamnopyranoside	Flavonol glycoside gallates	Leaves	[1]
Myricetin 3-O-(4″-O-galloyl)-α-rhamnopyranoside	Flavonol glycoside gallates	Leaves	[1]
Myricetin 3-O-(6″-O-galloyl)-β-galactopyranoside	Flavonol glycoside gallates	Leaves	[1]
Myricetin 3-O-β-D-glucoside	Flavone	Whole plant	[3]
Myricetin-3-O-α-L-rhamnoside	Flavone	Leaves	[4]
Quercetin	Flavonol	Whole plant	[1], [3], [4]
Quercetin-3-O-β-D-glucoside	Flavonoid	Whole plant	[3]
Quercetin 3-O-α-L-rhamnoside	Flavonoid	Leaves	[4]
Quercetin 3-O-α-rhamnopyranoside	Flavonol glycoside	Leaves, Roots	[1], [2]
Quercetin 3-O-(2″-O-galloyl)-α-rhamnopyranoside	Flavonol glycoside gallates	Leaves	[1]
Isoquercitin	Flavonoid	Whole plant	[5]
Isorhamnetin	Flavonol	Whole plant	[3]

Compound Name	Compound Type	Extract Part	Reference
Eriodictyol	Flavanone	Leaves	[1]
Homoeriodictyol	Flavanone	Leaves	[1]
Morin	Flavonol	Whole plant	[5]
Apigenin	Flavone	Leaves	[1]
Luteolin	Flavone	Leaves	[1]
Naringenin	Flavanone	Leaves	[1]
(-)-Epigallocatechin 3-gallate	Flavan-3-ol	Leaves, Roots	[1], [2]
Epigallocatechin 3-O-(3′-O-methyl)-gallate	Flavan-3-ol	Leaves	[1]
Epigallocatechin 3-O-(3′,5′-di-O-methyl)-gallate	Flavan-3-ol	Leaves	[1]
Isodihydrosyringetin	Flavanone	Roots	[2]
Kaempferol	Flavonol	Whole plant	[3]
Kaempferol-3-O-α-L-rhamnopyranoside	Flavonoid	Whole plant	[3]
(+)-Catechin	Flavan-3-ol	Whole plant	[3]
Isorhamnetin-3-rutinoside	Flavonoid-3-o-glycoside	Whole plant	[3]
Samarangenin B	Tannin	Roots	[2]
N-trans-caffeoyltyramine	Alkaloid	Roots	[2]
N-trans-feruloyltyramine	Alkaloid	Roots	[2]
Mannitol	Monosaccharide	Whole plant	[3]
LSP21	Polysaccharide	Roots	[6]

Compound Name	Compound Type	Extract Part	Reference
β-sitosterol	Sterol	Whole plant	[3]
Oleanolic acid	Pentacyclic triterpenoid	Whole plant	[3]
Ethyl gallate	Phenolic acid	Whole plant	[3]
ursolic acid	pentacyclic triterpene acid		[1]
Gallic acid	Phenolic acid	Leaves, Roots	[1], [2]

References

1. Lin, L.C. and C.J. Chou, *Flavonoids and phenolics from Limonium sinense.* Planta Med, 2000. **66**(4): p. 382-3.
2. Lin, L.C., Y.C. Kuo, and C.J. Chou, *Anti-herpes simplex virus type-1 flavonoids and a new flavanone from the root of Limonium sinense.* Planta Med, 2000. **66**(4): p. 333-6.
3. Liu, X., *Chemical constituents of Limonium sinense.* Chinese Traditional and Herbal Drugs., 2011. **42**(02): p. 230-233.
4. Hongzhu, G. and Y. Jiurong, *Studies on the Chemical Constituents of Chinese Sealavender (Limonium sinense).* Chinese Traditional and Herbal Drugs, 1994.
5. Fan, Y., *Simultaneous determination of isoquercetin,morin,quercetin,luteolin and apigenin in Limonium sinense (Girard) Kuntze by RP-HPLC.* Chinese Journal of Pharmaceutical Analysis, 2014. **34**(04): p. 632-635.
6. Tang, X.H., et al., *Isolation and identification of anti-tumor polysaccharide LSP21 from Limonium sinense (Girard) Kuntze.* Int J Biol Macromol, 2014. **70**: p. 138-42.
