# Supplementary material for: Integrated network pharmacology and cellular assay reveal the biological mechanisms of Limonium sinense (Girard) Kuntze against breast cancer
Source: BMC Complement Med Ther. Author manuscript; Available in PMC 2023 Nov 21. (PMC10644419; doi:10.1186/s12906-023-04233-z)
Supplement: Table S13 [file EMS189939-supplement-Table_S13.pdf]

Supplementary Table 13. Details of the GEO datasets collected for Apigenin treatment

Datasets	Title	Cells	Platform	Induced sample information
GSE119552 [1]	Effect of estradiol, zearalenone and apigenin on ER-positive breast cancer cells MCF-7	MCF7 cells	Agilent-039494 SurePrint G3 Human GE v2 8x60K Microarray 039381 (Feature Number version)	MCF-7_Control: 4<br>MCF-7_Apigenin: 4
GSE120550 [2]	Transcriptomic profiling of MDA-MB-231 cells treated with TNFα [40 ng/ml] ± Apigenin [40 μM]	MDA-MB-231 cells	[HuGene-2_1-st] Affymetrix Human Gene 2.1 ST Array [transcript (gene) version]	MDA-MB-231_Control: 3<br>MDA-MB-231_Apigenin: 3

References

1. Lecomte, S., et al., *Deciphering the Molecular Mechanisms Sustaining the Estrogenic Activity of the Two Major Dietary Compounds Zearalenone and Apigenin in ER-Positive Breast Cancer Cell Lines.* Nutrients, 2019. **11**(2).
2. Bauer, D., E. Mazzio, and K.F.A. Soliman, *Whole Transcriptomic Analysis of Apigenin on TNFalpha Immuno-activated MDA-MB-231 Breast Cancer Cells.* Cancer Genomics Proteomics, 2019. **16**(6): p. 421-431.
